# Low-Scaling, Efficient and Memory Optimized Computation of Nuclear Magnetic Resonance Shieldings within the Random Phase Approximation Using Cholesky-Decomposed Densities and an Attenuated Coulomb Metric

**DOI:** 10.1021/acs.jpca.4c02773

**Published:** 2024-09-06

**Authors:** Viktoria Drontschenko, Christian Ochsenfeld

**Affiliations:** † Chair of Theoretical Chemistry, Department of Chemistry, University of Munich (LMU), D-81377 Munich, Germany; ‡ Max Planck Institute for Solid State Research, D-70569 Stuttgart, Germany

## Abstract

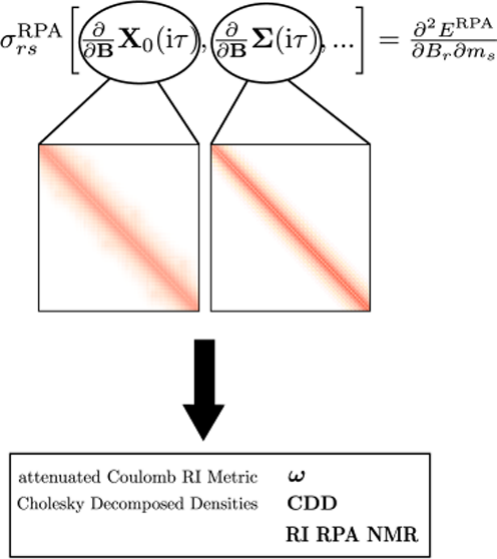

An efficient method for the computation of nuclear magnetic resonance (NMR) shielding tensors within the random phase approximation (RPA) is presented based on our recently introduced resolution-of-the-identity (RI) atomic orbital RPA NMR method [DrontschenkoV.J. Chem. Theory Comput.2023, 19, 7542–755437863033 10.1021/acs.jctc.3c00542] utilizing Cholesky decomposed density type matrices and employing an attenuated Coulomb RI metric. The introduced sparsity is efficiently exploited using sparse matrix algebra. This allows for an efficient and low-scaling computation of RPA NMR shielding tensors. Furthermore, we introduce a batching method for the computation of memory demanding intermediates that accounts for their sparsity. This extends the applicability of our method to even larger systems that would have been out of reach before, such as, e.g., a DNA strand with 260 atoms and 3408 atomic orbital basis functions.

## 1 Introduction

The accurate and efficient prediction of nuclear magnetic resonance (NMR) shielding tensors from quantum chemical calculations has emerged as an important technique to assist experimental NMR spectroscopy in structure determination.^1−7^ Methods for the computation of NMR shielding tensors providing reasonable accuracy at moderate computational cost include Hartree–Fock (HF)^8−12^ theory and density functional theory (DFT).^13−15^ Here, the development of low-scaling methods allowed the computation of systems with over 1000 atoms.^16,17^ In general a higher level of accuracy can be achieved by wave function based post HF-methods such as Møller–Plesset perturbation theory (MP2),^18,19^ multiconfigurational self-consistent field (MCSCF) methods,^20^ and coupled cluster (CC) variants.^21−23^ MP2 has been shown to be more accurate than HF and DFT,^19,24,25^ while coupled cluster singles and doubles (CCSD) as well as CCSD with additional perturbative triples (CCSD(T)) are among the most accurate methods.^26^ However, the increased accuracy comes at an increased computational cost, which makes the development of efficient and low-scaling techniques an important task in the development of wave function based NMR methods. Specifically, much progress has been made in this regard for MP2 and its related methods.^27−36^

A method that has recently been shown to combine both accuracy and low computational cost is the random phase approximation (RPA). In a recent benchmark study it was shown that RPA based on a HF reference calculation is able to provide NMR shielding tensors comparable in accuracy to CCSD.^37^ Due to these promising results, we successfully derived and implemented a method for the computation of analytical RPA NMR shielding tensors.^38^

RPA is usually implemented as a post-Kohn–Sham (KS)^39^ method. It stands on the fifth rung on Jacob’s ladder^40^ of density functional approximations, and does not contain any empirical parameters. The RPA ground state energy can be obtained within the framework of DFT^39,41^ by applying the adiabatic-connection fluctuation–dissipation theorem (ACFDT).^42−44^ The ACDFT provides an exact expression for the electron correlation energy in terms of the KS response function and the response function of the system of fully interacting electrons. However, the latter quantity contains the exchange-correlation kernel, the functional derivative of the exchange-correlation potential with respect to the density, which is not known. The simplest approximation is to neglect this contribution, which leads to the (direct) random phase approximation. From its formal derivation, RPA is able to seamlessly incorporate the description of mid- to long-range dispersion interactions, eliminating the need for empirical corrections, and, furthermore, it can also be employed for metallic systems.^45−48^ However, in its original form, the computation of RPA energies scales as  with the system size *M*, limiting its applicability to small systems. Furche and co-workers extended the applicability of RPA by utilizing the resolution-of-the-identity (RI)^49^ approximation achieving an  scaling, which makes RPA one of the formally lowest scaling correlation methods. Further, by reformulating the RPA ground state energy expression in the atomic orbital (AO) basis^50^ and using an attenuated Coulomb RI metric,^51^ Cholesky decomposed ground state densities,^52^ and sparse matrix algebra we were able to obtain asymptotically linear scaling with the system size. These techniques were also applied to the computation of first-order properties within RPA, specifically analytical nuclear gradients, thereby achieving an  scaling.^53^

In ref (38) we introduced a method for the calculation of analytical RPA NMR shielding tensors for the first time. We used an atomic orbital formalism as well as the RI approximation with the Coulomb RI metric. This provides an optimal starting point to improve the computational efficiency as well as scaling behavior. In this work we switch to the attenuated Coulomb RI metric, thereby introducing sparsity in the three-center integral tensors, and use Cholesky decomposition (CD) of ground state densities as well as CD of the Green’s function in the positive imaginary time domain. The introduced sparsity is efficiently exploited using sparse matrix algebra.

While these techniques are able to improve the computational efficiency of the method, another challenge has to be addressed. For the computation of NMR shielding tensors, the three-center RI tensors as well as their **B**-field derivatives have to be stored in memory. Together with the memory requirements of intermediates arising during the calculation, the memory required for the method easily exceeds the available random access memory (RAM), which limits the tractable system sizes. For the computation of RPA energies, we solved this problem in ref (54) by developing an optimized batching scheme for the computation of the response function by batching over auxiliary function indices, atomic orbital indices, as well as time quadrature points. The three-center integral tensor was recomputed for each batch (integral-direct) and transformed on the fly. The optimal number of batches was computed by minimizing the number of integral calculations, under the constraint of not exceeding the system memory using a Lagrange formalism. This constitutes the best trade off between program runtime and memory demand. However, the optimized batching was so far implemented only for dense matrices and, thus, the sparsity of matrices was not exploited when computing the number of batches. When utilizing sparse matrices, the challenge lies in approximating their memory demand, which is not known beforehand and only determined at program runtime. In this context, we want to note that the most common batching approach within electron correlation methods in literature is batching over one index only, such as the auxiliary function index,^52,76−78^ AO index,^79,80^ or molecular orbital index^81,82^ (virtual or occupied). The number of batches is chosen to be as low as possible without exceeding the available memory. Since dense matrices are employed, the memory demand can be easily approximated.

In this work, we introduce a sparse batching method by approximating the memory demand of sparse matrices by sampling the auxiliary function space and precomputing a number of intermediates. As will be demonstrated, the overhead for the precomputations is practically insignificant. Since there is a considerable number of intermediates within RPA NMR that have to be computed by batching, we opted for a simple batching scheme over auxiliary function indices. The three-center integrals and their magnetic field derivatives are stored on disk and read into memory for each batch and transformed on the fly. While this batching scheme is not optimal yet, it constitutes a starting point for the development of sparse batching methods. In a next step, our sparse sample batching method could be combined with optimal batching, however, we leave this for future work.

The present work is structured as follows: We start with a brief review of the atomic orbital RI-RPA-NMR method in Section 2.2 and continue in Section 2.3 with the description of our new ω-CDD-RI-RPA-NMR method. The theory is concluded in Section 2.4 with the description of our new batching method to achieve a memory efficient implementation. Next, after establishing the computational details in Section 3, we start the results section by considering the accuracy of the introduced approximations in Section 4.1. The scaling is analyzed in Section 4.2. In Section 4.3 the performance of our method is examined, by considering the timings for the most computationally demanding steps within ω-CDD-RI-RPA-NMR (Section 4.3.1) and analyzing the batching in detail for sparse systems in Section 4.3.2 and in Section 4.3.3 for dense systems that are more representative of potential applications. Finally, the conclusion is given in Section 5.

## 2 Theory

The NMR shielding tensor **σ**^*A*^ of a nucleus *A* is given by the mixed second derivative of the electronic Energy *E* with respect to the components of the nuclear magnetic moment **m**^*A*^ and the magnetic field **B** evaluated at zero
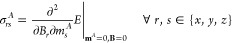
1In this work, we will compute the NMR shielding tensor at the RPA level of theory: we start by introducing the notation used throughout this work and subsequently, in Section 2.2, we give a short summary of the RPA NMR method introduced in ref (38). The theory for the low-scaling RPA NMR method is detailed in Section 2.3 and memory efficient batching implementation is provided in Section 2.4.

### 2.1 Notation

The following notation is used in this work:

- μ, ν, λ, σ: Atomic orbitals (total number: *N*).
- *P*, *Q*, *R*, *S*: Auxiliary functions (total number: *N*_aux_).
- *i*, *j*: Occupied Cholesky orbitals (total number: *N*_occ_).
- *a*, *b*: Virtual Cholesky orbitals (total number: *N*_virt_(iτ)).

Mulliken notation is used for two- and three-center integrals. Einstein’s sum convention is employed.^55^ The derivative of a quantity *O* with respect to a perturbation ξ, i.e., , is abbreviated as *O*^ξ^.

### 2.2 Atomic Orbital RI-RPA Nuclear Magnetic Resonance Shieldings

#### 2.2.1 AO-RI-RPA Total Energy

The total energy of the electronic ground state can be expressed within the adiabatic-connection formalism^43^ as^42,44^
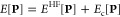
2where the Hartree–Fock energy *E*^HF^ and the correlation energy *E*_c_ are evaluated with the density matrix **P** from a preceding KS-DFT or HF calculation. Further, by applying the zero-temperature fluctuation–dissipation theorem, the random phase approximation,^56^ as well as the RI approximation,^57−59^ the correlation energy can be expressed as
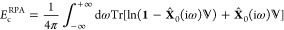
3The RI approximation employed in the above equation allows to factorize four-center-two-electron integrals within an arbitrary metric *m*_12_ as
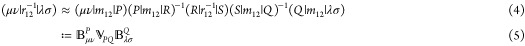
leaving behind three-center integral tensors  as well as two-center integral tensors summarized in the electron–electron interaction operator , both defined as
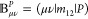
6
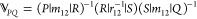
7Please note that matrix operations are to be taken before indexing in this work. The noninteracting response function in the imaginary frequency domain **X̂**_0_(iω) in eq 3 is obtained by the Fourier transform
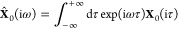
8which simplifies to a cosine transform,^52,60^ or, equivalently to a double Laplace transform^50,51^ in case **X**_0_(iτ) is an even function in the imaginary time domain. The response function in the imaginary time domain is given by^61^
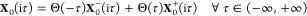
9
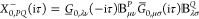
10
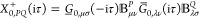
11with the noninteracting Green’s function defined as
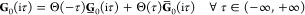
12
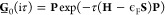
13
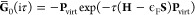
14here ϵ_F_ denotes the Fermi level,^51,62^ Θ(τ) the Heaviside step function, and **S** is the overlap matrix. The occupied and virtual density matrix is given by **P** and **P**_virt_, respectively. The Hamiltonian **H** is defined according to
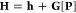
15
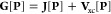
16with the matrix representation of the one-electron Hamiltonian **h**, the Coulomb potential **J**, and exchange-correlation potential **V**_xc_. Computing the response function in the imaginary time domain and Fourier transforming into the imaginary frequency domain (rather than direct computation in the (iω)-domain) allows for an atomic orbital formulation opening the way for linear scaling RPA implementations.^50−52^

#### 2.2.2 First Derivative with Respect to the Nuclear Magnetic Moment

The first derivative of the total RPA energy, i.e., eq 2, with respect to the nuclear magnetic moment **m** is given by^38,53^
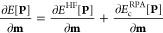
17The derivative of the HF energy with respect to **m** can be expressed as
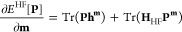
18
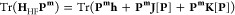
19with the matrix representation of the Hartree–Fock exchange **K**. Since the HF energy is not stationary with respect to the KS density matrix, the response **P**^**m**^ has to be evaluated, which differs from regular HF gradient calculations where it can be avoided.^63^

Next, differentiating the RPA correlation energy given in eq 3 with respect to **m** results in^38,53^
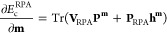
20It is important to note that the density matrix response **P**_virt_^**m**^ could be avoided in this expression using the relation **P**_virt_^**m**^ = −**P**^**m**^.^38,53,64^

The central intermediates **V**_RPA_ and **P**_RPA_ are defined as
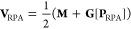
21
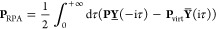
22Further, **M** is given by
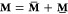
23
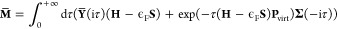
24
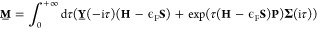
25and **Y**(iτ) is expressed according to
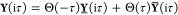
26
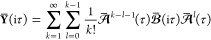
27
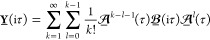
28with  defined as
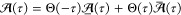
29
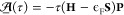
30
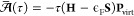
31and  as
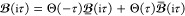
32
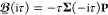
33
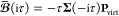
34The **Y** matrices are most efficiently evaluated using a recursion scheme. The detailed procedure and corresponding equations can be found in refs (34 and 64).

Furthermore, the correlated self-energy **Σ**(iτ), which is of central importance in this work, is defined as
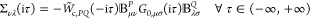
35with the intermediate
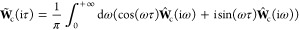
36defined in terms of the correlated screened Coulomb interaction
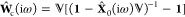
37

#### 2.2.3 Second Derivative with Respect to the Magnetic Field: NMR Shielding Tensor

The NMR shielding tensor, i.e., the second mixed derivative of the total RPA energy with respect to **m** and **B**, is defined as^38^
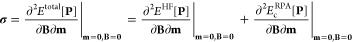
38Evaluating the above equation by forming the second derivative of the HF energy and of the RPA correlation energy with respect to **m** and **B** results in^38^
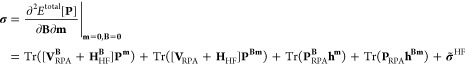
39
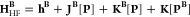
40It should be noted that the term **J**[**P**^**B**^] in the above equation is zero, due to the skew-symmetry of the purely imaginary density matrix derivative and the contribution **σ̃**^HF^ = Tr(**P**^**B**^**h**^**m**^) + Tr(**Ph**^**Bm**^) is treated separately with the usual techniques used for the computation of HF and DFT shifts for simplicity. The perturbed density matrix **P**^**m**^, given in the first term of eq 39, can, in principle, be computed by solving the coupled perturbed KS (CPKS) equations for all perturbations of the nuclear magnetic moment. However, a more efficient route is to use the Z-vector technique,^64,65^ which requires the solution of only one CPKS equation. We employ the density matrix-based Laplace-transformed CPKS method^66^ developed by our group within our AO formulation.

At this point, the challenging terms from eq 39 that remain to be evaluated are the **B**-field derivatives of the RPA intermediates **V**_RPA_ and **P**_RPA_ as well as the second derivative of the density matrix **P**^**Bm**^. In this context the second derivative of the density matrix, **P**^**Bm**^, can be computed using a nested Z-vector approach^32,34^ which was introduced in the framework of Laplace-transformed MP2 NMR. Employing the efficient approach of ref (34) allows to derive the final equations for the computation the NMR shielding tensor within RPA (in terms of **V**_RPA_^**B**^ and **P**_RPA_^**B**^) as detailed in ref (38). The central task in RPA NMR is, however, the evaluation of **V**_RPA_^**B**^ and **P**_RPA_^**B**^, which is the subject of the next section.

#### 2.2.4 Computation of RPA NMR Intermediates: **V**_RPA_^**B**^ and **P**_RPA_^**B**^

**Figure 1:**
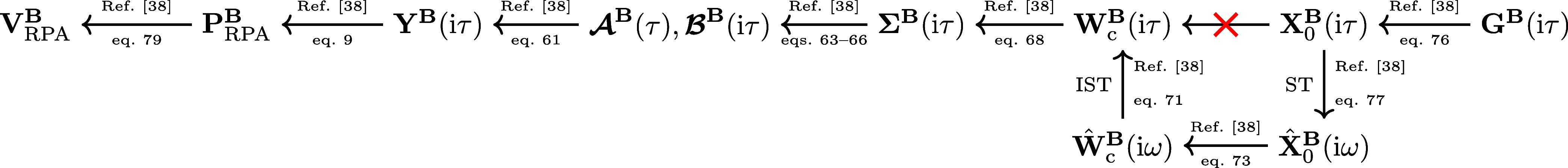
Schematic representation of the derivation of the intermediates **V**_RPA_^**B**^ and **P**_RPA_^**B**^. ST denotes a sine transform and IST an inverse sine transform. All arrows are labeled with the corresponding equations from ref (38). (Reproduced from ref (38). Copyright 2023 American Chemical Society).

The detailed evaluation of the intermediates **V**_RPA_^**B**^ and **P**_RPA_^**B**^ has been presented in ref (38) and we refer to this reference for a complete derivation and the corresponding equations. Figure 1 provides an overview over the important intermediates that have to be differentiated in order to obtain **V**_RPA_^**B**^ and **P**_RPA_^**B**^. The equation numbers from ref (38) corresponding to the specific steps are provided and we intend to only review the detailed equations for the two most demanding steps, specifically, the computation of **Σ**^**B**^(iτ) and **X**_0_^**B**^(iτ).

The **B**-field derivative of the self-energy can be obtained by either directly differentiating eq 35 using the product rule, as has been done in ref (38), or by taking the partial derivatives of **Σ**(iτ) with respect to **W̃**_c_(iτ), , and **G**_0_(iτ) multiplied by the respective **B**-field derivative of each quantity. This results in
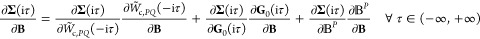
41For the **B**-field derivative of the self-energy in the positive imaginary time domain it follows for the partial derivatives of eq 41 (for τ > 0)
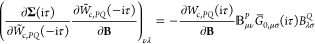
42
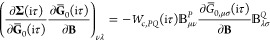
43
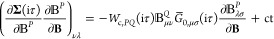
44In eq 44 the abbreviation “ct” denotes the conjugate transpose of the first term on the right-hand site, a notation that will be employed from now on. The terms **W**_c_(iτ) and **W**_c_^**B**^(iτ) are given by the cosine and sine transforms, respectively, as
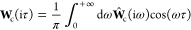
45
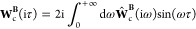
46

The partial derivatives for the self-energy in the negative imaginary time domain are given for τ ≥ 0 by
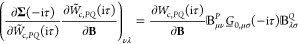
47
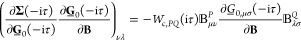
48
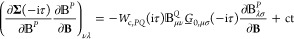
49

The **B**-field derivative of the response function, i.e., eq 11, can be obtained using the same strategy yielding
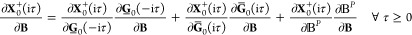
50with the partial derivatives
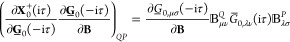
51
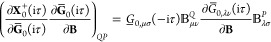
52
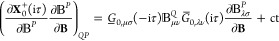
53

The self-energy and response function as well as their **B**-field derivatives can be computed with a formal scaling of  and thus are the steepest scaling steps in the computation of RPA NMR shieldings. Further, since these steps require the three-center integrals as well as their **B**-field derivatives, they are also the most demanding steps in terms of memory requirements. Thus, for an efficient implementation of RPA NMR it is necessary to optimize these steps in terms of computational effort as well as memory requirements. The former is described in the next section, where we employ a local RI metric and Cholesky decomposition together with sparse matrix algebra to reduce the computational effort as well as lower the scaling. Then, in Section 2.4, those computational optimizations are combined with a batching scheme to achieve a memory efficient implementation.

### 2.3 Low-Scaling RPA NMR Method: ω-CDD-RI-RPA-NMR

#### 2.3.1 Strategies for Low-Scaling: Local RI Metric and Cholesky Decomposition of Density Type Matrices

The computation of the response function and self-energy as well as their respective **B**-field derivatives constitute the most computationally demanding steps in the calculation of RPA NMR shieldings. In this section we describe several methods to optimize these steps and lower their scaling.

As mentioned in Section 2.2.1 we employ the RI approximation which allows to avoid the four-center-two-electron integrals and instead work with lower rank tensors, specifically three-center and two-center integral tensors (see eqs 4 and 5). The crucial factor to decrease computational effort and lower the scaling in extended molecular systems is the choice of the RI metric *m*_12_. The Coulomb metric  has proven to be optimal for modeling density type repulsions.^67^ However, due to the very slow decay, it couples the charge distributions (μν) with the auxiliary functions *P* in the three-center integral tensor (μν|*m*_12_|*P*) over effectively infinite distances. Thus, no sparsity can be gained in their matrix representation. In contrast to that, the overlap metric *m*_12_ = δ(*r*_12_) is very local since it decays as exp(−*r*_12_^2^) for Gaussian basis sets. The introduced sparsity comes, however, at the cost of decreased accuracy.^67^ A metric that combines both, accuracy and sparsity, is the Coulomb metric attenuated by the complementary error function (erfc)^51,68,69^ expressed as
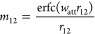
54The attenuation parameter *w*_att_ determines the attenuation strength. By varying this parameter the sparsity and loss in accuracy can be controlled. In the limiting cases of *w*_att_ → 0 and *w*_att_ → ∞ the Coulomb metric and overlap metric are retrieved, respectively. It has been shown in ref (51) that the attenuation parameter *w*_att_ = 0.1 au gives very good results for RPA energy calculations by balancing accuracy and sparsity. Further, ref (53) reports the same for RPA nuclear gradients. In this work we will further investigate if these findings can be extended to second order properties, specifically RPA NMR shieldings.

Another strategy we will employ, is pivoted Cholesky decomposition (CD)^50−52,70−74^ of density type matrices. In this context the pivoted Cholesky decomposition of a positive semidefinite (*N*_R_ × *N*_R_) matrix  is given by
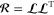
55where  is a lower triangular matrix with dimensions . Thus, if the rank of a matrix is significantly less than its dimensions then substantial savings in computational effort and memory requirements can be achieved. In order to make use of that, we start by considering that the Green’s function in the negative imaginary time domain is invariant to projection onto the occupied space, which gives rise to the Cholesky decomposition of the occupied ground state density matrix **P** = **LL**^T^ resulting in^52^
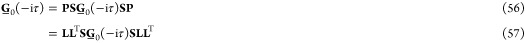
where the Cholesky matrix **L** has dimensions (*N* × *N*_occ_). It should be noted that the CD of the virtual density matrix is of not much use since its rank corresponds to the number of virtual orbitals *N*_virt_, which is not significantly less than the number of basis functions. Next, the CD of the Green’s function in the positive imaginary time domain is considered according to
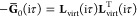
58Since **G̅**_0_(iτ) is a negative semidefinite matrix it is made positive semidefinite by multiplication with −1. The rank of **G̅**_0_(iτ), which corresponds to the columns of **L**_virt_(iτ), is time dependent and decreases with increasing time, that is rank(**G̅**_0_(iτ)) ≤ *N*_virt_.

It is important to note that CD of the **B**-field derivative of the Green’s function and density matrix is not possible since both matrices are not positive semidefinite.

#### 2.3.2 Calculation of the Self-Energy and Its **B**-Field Derivative

For the calculation of the self-energy given by eq 35 we can insert eq 58 for the computation of **Σ**(iτ) and eq 57 for the computation of **Σ**(−iτ) leading to
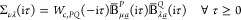
59
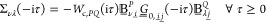
60where we have used the fact that the unperturbed correlated screened Coulomb interaction is an even function in the (iτ)-space. Further, the following notation for transformed quantities has been introduced
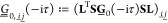
61
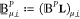
62
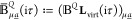
63

Next, for the **B**-field derivative of the self-energy we start by considering the partial derivatives of the self-energy in the positive imaginary time domain. For the partial derivative with respect to **W̃**_c_(iτ), i.e., eq 42, and for the partial derivative with respect to the three-center integrals, i.e., eq 44, we can employ eq 55 leading to
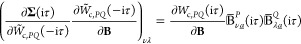
64
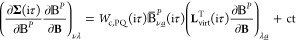
65For the term containing the derivative of the Green’s function, that is eq 43, CD cannot be used since **G**_0_^**B**^ is not positive semidefinite.

Similarly, for the self-energy in the negative imaginary time domain eq 56 can be inserted into the partial derivative term containing **W**_c_^**B**^(iτ), i.e., eq 47, and the term containing , i.e., eq 49, resulting in
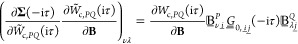
66
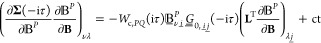
67Again, the term containing the derivative of the Green’s function eq 48 cannot be transformed using CD.

#### 2.3.3 Calculation of the Response Function and its **B**-Field Derivative

The evaluation of the response function can be restricted to positive imaginary times, that is eq 11, since the unperturbed response function is an even function in the (iτ)-domain. Using eqs 56 and 58 allows to express the response function according to
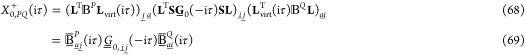
where
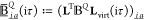
70

For the **B**-field derivative of the response function eq 50 its partial derivatives given by eqs 51–53 can be considered. Inserting eq 56 into eq 52 and into eq 53; furthermore inserting eq 58 into eq 51 and into eq 53 yields
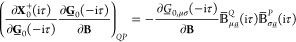
71
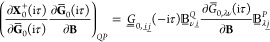
72
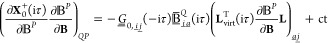
73

#### 2.3.4 Scaling

When discussing the scaling in this section, we refer to the asymptotic scaling in the limit of very large system sizes. In this context a distinction is made for the theoretical scaling of the ω-CDD-RI-RPA method employing dense matrix algebra, which is generally denoted as “formal scaling” and the method is referred to as “dense method”, and the same method employing sparse matrix algebra which is discussed in the context of computing sparse systems, which is denoted as “sparse method”.

As noted earlier, our AO-RI-RPA-NMR implementation of ref (38) has a formal scaling of  and scales as  with the system size. In this section we investigate the asymptotic scaling of the dense and sparse implementation for the computation of the self-energy and response function as well as their **B**-field derivatives using our new ω-CDD-RI-RPA-NMR formulation as introduced in the previous sections. The results are shown in Table 1. The asymptotic scaling of the sparse method for the response function and its **B**-field derivative can be reduced to linear provided that the matrices **G**_0_(iτ) and  as well as their **B**-field derivatives are sparse. The asymptotic scaling for the self-energy and its **B**-field derivative can be reduced only to quadratic at this stage, since the correlated screened Coulomb interaction is in general a dense matrix as it contains the  term.^53^ Thus, for sparse systems generally a quadratic scaling with the system size would be expected for the ω-CDD-RI-RPA-NMR method.

**Table 1:** Asymptotic Scaling for the Computation of the Self-Energy and Response Function As Well As Their **B**-Field Derivatives within the ω-CDD-RI-RPA-NMR Method Utilizing Dense Matrix Algebra (Dense) and Using Sparse Matrix Algebra (Sparse) Assuming Sparse Systems^a^

		scaling	
quantity	equation nr.	dense	sparse
**Σ**(iτ)	59		
**Σ**^**B**^(iτ)			
	64		
	43		
	65		
**Σ**(−iτ)	60		
**Σ**^**B**^(−iτ)			
	66		
	48		
	67		
**X**_0_(iτ)	69		
**X**_0_^**B**^(iτ)			
	71		
	72		
	73		

a For the **B**-field derivatives, the scaling for all partial derivative terms is provided. Further, the equation numbers for each term are noted.

### 2.4 Memory Efficient Implementation

As a starting point for the implementation of our new ω-CDD-RI-RPA-NMR method, we used the framework of our AO-RI-RPA-NMR method.^38^ An overview containing all steps of the calculation is provided in the Supporting Information of ref (38). However, the steps involving the computation of the response function and self-energy as well as their **B**-field derivatives will be replaced by the memory efficient method introduced below. We employ the following directives for a memory efficient implementation:

- **Σ**(iτ), **X**_0_(iτ), **Σ**^**B**^(iτ), and **X**_0_^**B**^(iτ) are computed for one τ quadrature point at a time.
- Each partial derivative term corresponding to **Σ**^**B**^(iτ), and **X**_0_^**B**^(iτ) is computed within a separate batching scheme.
- The partial derivative terms for the magnetic field derivative of the self-energy are computed for the positive and negative imaginary time domain within the same batching scheme.
- The three-center integrals and their **B**-field derivatives are stored on disk in a compact matrix format, where only elements (with a significant contribution) from the upper triangular matrix are stored for each auxiliary function. When the integrals and their derivatives are needed within the calculation they are copied back into a regular matrix format for one auxiliary function at a time and the lower triangular matrix is filled by considering the symmetry of the three-center integrals and the skew-symmetry of its **B**-field derivative.
- Intermediates that are stored on disk are read into memory for one aux-batch for efficient read performance.
- Integrals are transformed for one auxiliary function at a time.
- For efficiency, common intermediates are precomputed and stored on disk to be reused.
- The loops over auxiliary function indices are parallelized.

With these guidelines in mind, a memory efficient implementation for the computation of **Σ**(iτ) and **X**_0_(iτ) as well as their magnetic field derivatives is possible. The detailed algorithms and technical details of the implementations are provided in the Supporting Information.

#### 2.4.1 Computation of Batches: Accounting for Sparsity

In the previous section, batching schemes were described for the computation of the self-energy and response function as well as their **B**-field derivatives. The task ahead is to develop a method for the computation of the number of batches by accounting for the sparsity of all matrices within a step of the calculation. In this context a general framework for the computation of batches will be presented.

**Figure 2:**
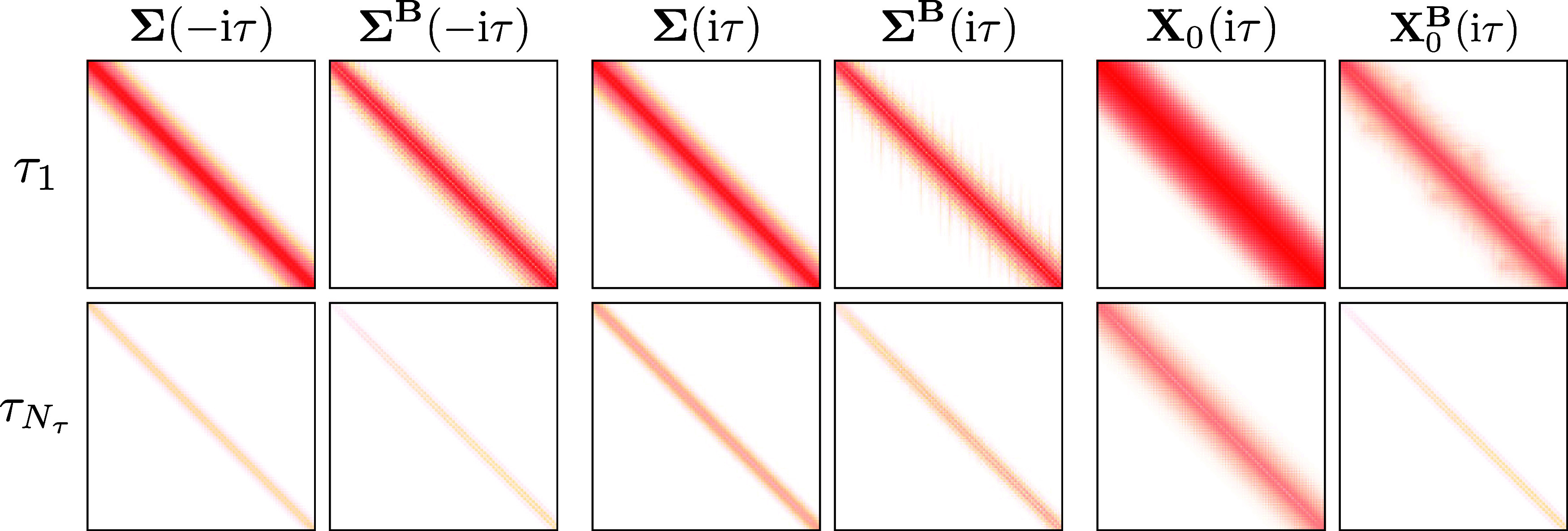
Sparsity patterns of the self-energy and the response function as well as their **B**-field derivatives (for one direction of the magnetic field) for the first time grid point (τ_1_) and the last one (τ_*N*_τ__) for the linear alkane C_80_H_162_ using the cc-pwCVDZ basis set with the corresponding RI basis set.

First, the dependence of the sparsity on the time quadrature point τ is investigated for the self-energy, response function, and their **B**-field derivatives. In Figure 2 the sparsity patterns for all of the mentioned quantities are shown for the linear alkane C_80_H_162_ in a cc-pwCVDZ basis set. As can be seen, the sparsity shows a strong dependence on the τ quadrature point for all quantities, increasing with the value of τ. Presumably, this can be traced back to the exponential functions within the Green’s functions eqs 13 and14 which decay with increasing τ. This indicates that the sparsity associated with this exponential decay is present in both, sparse and dense systems. Due to this observation, the number of batches is recomputed for each τ quadrature point.

Next, an approximation for the memory demands of sparse matrices is necessary, since they are not previously known and only determined at program runtime. In our implementation sparse matrices are implemented in a block-sparse format, where the matrices are divided into blocks of constant size. Upon allocation of the sparse matrix, the blocks are screened. Each block with an L2-norm lower than a threshold value ϑ_a_ is removed. A second screening threshold ϑ_m_ is used for the matrix–matrix multiplication. If the product of the L2-norms of two matrix-blocks is below ϑ_m_ the matrix–matrix multiplication of those blocks is not preformed. More information about block-sparse matrices, their matrix–matrix multiplication routine, as well as the memory allocation technique is given in the Supporting Information of ref (75).

For the computation of batches the relevant memory demands that need to be approximated are those of third-order tensors  with dimension (*N*_*k*_ × *N*_*l*_ × *N*_*m*_). In our implementation third-order tensors are generally represented by a vector containing a number of *N*_*m*_ sparse matrices of size (*N*_*k*_ × *N*_*l*_). Thus, the total memory demand of the tensor is given by the sum of the memory demands of the respective sparse matrix associated with each *m*. Specifically in this work the third index *m* refers to the auxiliary function index. To approximate the total memory demand of these tensors, we sample the auxiliary function space by precomputing intermediate quantities for a number of auxiliary functions to determine their memory demands. Subsequently, the average memory demand for one auxiliary function is computed from the sampling and multiplied by the total number of auxiliary functions to approximate the memory demands of the tensor. Further, for **B**-field derivatives of third order tensors, we carry out the sampling for all **B**-field directions, compute the approximate memory demands, and determine the maximum memory out of all **B**-field directions. The maximum memory is then used for the computation of batches. In this work, we sample the auxiliary function space in steps of 100, which provides reliable results while not significantly increasing the computational effort of the method. Thus, together with the available system memory, the number of batches can be easily determined. By recomputing the batches for each τ quadrature point and approximating the sparsity of all matrices as described, we are able to account for sparsity in the computation of the number of batches for each batching scheme.

## 3 Computational Details

Our new method was implemented in the FermiONs++ program package.^83−85^ The RPA NMR shieldings computed in this work are based on preceding Hartree–Fock calculations. In ref (37) this setup was shown to provide accurate NMR shieldings of about CCSD quality. Optimized minimax grids^60^ for the time and frequency integration^52,60^ as well as the cosine and sine^86^ transformation are employed with 15 grid points, which has been shown in ref (38) to yield accurate results. For our new method, in the following denoted as ω-CDD-RI-RPA-NMR, we employ sparse matrix algebra (ϑ_a_ = 10^–7^, ϑ_m_ = 10^–9^, block size (96 × 96)) and the attenuated Coulomb metric with the attenuation parameter ω_att_ = 0.1 au. The truncation tolerance used for the pivoted Cholesky decomposition is 10^–11^. The implementation introduced in ref (38) is denoted as AO-RI-RPA-NMR and utilizes the Coulomb metric as well as dense matrix algebra routines as provided by the Math Kernel Library (version 2022.0.0). The frozen core approximation is not applied. The atomic orbital basis sets cc-pwCVDZ^87^ and cc-pwCVTZ^87^ were used with their corresponding RI basis set.^88^

## 4 Results and Discussion

### 4.1 Accuracy

Several techniques have been employed to improve the efficiency and the scaling of our RPA NMR method.^38^ To test the accuracy of our ω-CDD-RI-RPA-NMR method, calculations have been performed for the molecules in the test set assembled by Gauss and co-workers,^89^ excluding the molecules SO_2_ and O_3_ as has been done in ref (89) as well as PN as done in ref (38). Further, the test set of Flaig et al.^24^ was used. To analyze the deviations for more extended systems the monomers in the L7 test set^90^ (excluding C3GC monomer B and C2PD monomer A due to the high computing demands of the AO-RI-RPA-NMR method) as well as a set of three linear alkanes with 10–30 carbon atoms have been computed. NMR shieldings computed using the new ω-CDD-RI-RPA-NMR method are compared to the results obtained with the AO-RI-RPA-NMR method,^38^ which employs the Coulomb RI metric and dense matrix algebra. By this comparison, the error introduced through the local RI metric, the utilization of sparse matrix algebra, as well as Cholesky decomposition can be assessed for the ω-CDD-RI-RPA-NMR method. The cc-pwCVDZ and cc-pwCVTZ basis sets were used with the corresponding RI basis sets for the Gauss and Flaig test set. For the L7 test set and the set of linear alkanes the cc-pwCVDZ basis set was used due to the high computing demands of the AO-RI-RPA-NMR method that is used as the reference. The results are shown in Table 2, where the mean absolute errors (MAEs) and the standard deviations (SDs) of the ω-CDD-RI-RPA-NMR method compared to the AO-RI-RPA-NMR method are displayed for different test sets. As can be seen, the MAEs and SDs are on the order of 10^–3^ ppm for all considered test sets. Therefore, we can conclude that the introduced techniques do not compromise the accuracy of the method.

**Table 2:** MAEs (ppm) and SDs (ppm) of Isotropic NMR Shielding Constants Obtained Using the ω-CDD-RI-RPA-NMR Method with Respect to the AO-RI-RPA-NMR Results for the Molecules in the Gauss Benchmark Set, the Flaig Benchmark Set, the L7 Test Set, and a Set of Linear Alkanes^a^

benchmark set	basis set	MAE [10^–3^ ppm]	SD [10^–3^ ppm]
Gauss	cc-pwCVDZ	2.40	4.9
	cc-pwCVTZ	0.90	1.54
Flaig	cc-pwCVDZ	1.42	3.04
	cc-pwCVTZ	1.04	2.98
L7	cc-pwCVDZ	1.34	2.6
linear alkanes	cc-pwCVDZ	0.59	0.91

a The cc-pwCVDZ and cc-pwCVTZ basis sets were used with the corresponding RI basis set.

Given that the ω-CDD-RI-RPA-NMR method does not introduce any significant error, it is preferred over the AO-RI-RPA-NMR method due to its superior computational efficiency. In this context dense matrix algebra may be employed for smaller systems, while sparse matrix algebra is more efficient for larger systems and especially sparse systems. As shown in the Supporting Information for linear alkanes, an early crossover for C_10_H_22_ with the dense method is observed.

### 4.2 Scaling Behavior with the System Size

To analyze the effective scaling of our method with the system size, we carried out calculations on linear alkanes of increasing length using the cc-pwCVDZ basis set with the corresponding RI basis set. This system was chosen as an optimal test case due to its local electronic structure and the ability to systematically increase the system size. The calculations were performed on a compute node with AMD EPYC 9334 processors with 128 threads, 1.5 TB of RAM, and 4.7 TB of disk space. No batching was employed for the calculations.

**Figure 3:**
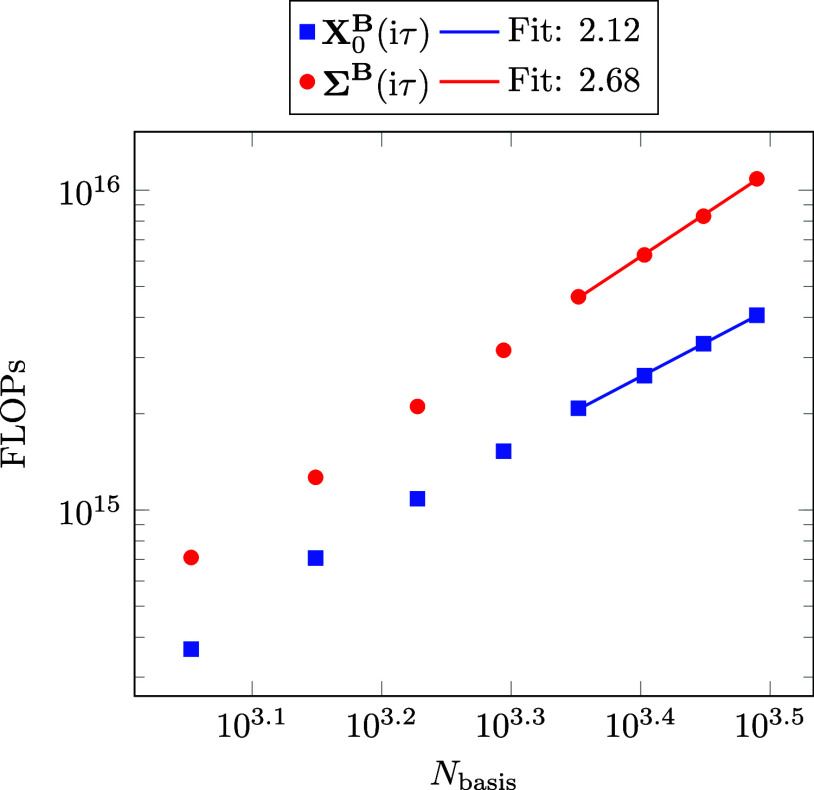
Log–log plot of the FLOPs for the calculation of **X**_0_^**B**^(iτ) (blue) and **Σ**^**B**^(iτ) (red) against the number of atomic orbital basis functions for linear alkanes of increasing size employing the ω-CDD-RI-RPA-NMR method.

The computation of the **B**-field derivative of the response function and the self-energy are the computationally most demanding steps and are among the formally steepest scaling steps of the calculation. Thus, to analyze the effective scaling behavior with the system size of both steps, we measure the number of floating point operations (FLOPs) that are required for their computation. The results are given in Figure 3. As can be seen, a quadratic scaling is obtained for the computation of **X**_0_^**B**^(iτ), which is higher than the expected asymptotic linear scaling that was discussed in Section 2.3.4. In comparison, in ref (52) an effective scaling of *M*^1.39^ was determined for the (non differentiated) response function by computing linear alkanes with 2170–7210 atomic orbital basis functions. Presumably, similar behavior could be obtained for the **B**-field derivative of the response function for larger systems. This would indicate a later onset of linear scaling behavior for larger systems. However, without batching the computation of ω-CDD-RI-RPA-NMR is limited to linear alkanes with up to 3090 atomic orbital basis functions on a compute node with 1.5 TB of RAM. A similar discussion can be applied to the computation of the **B**-field derivative of the self-energy. The observed scaling is also higher than the expected asymptotic quadratic scaling, which was discussed in Section 2.3.4. For the (non differentiated) self-energy an effective scaling of *M*^2.43^ was obtained in ref (53) using linear alkanes with 1930–4810 atomic orbital basis functions. Thus, we would expect to converge to the expected quadratic scaling for the **B**-field derivative of the self-energy for larger systems as well.

### 4.3 Performance

#### 4.3.1 Timings

**Figure 4:**
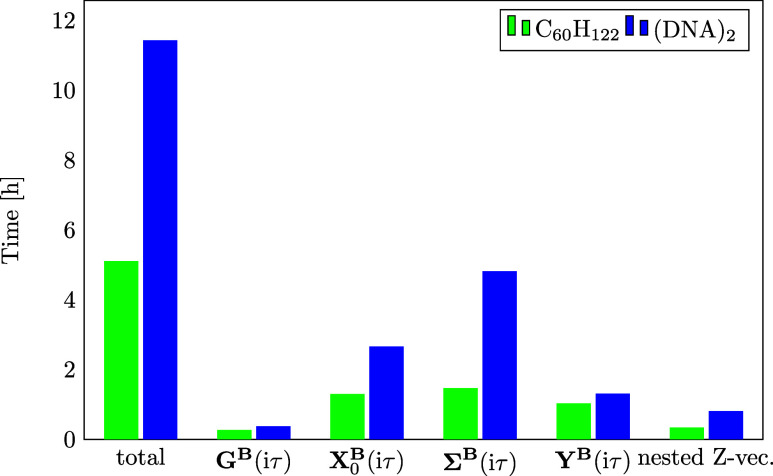
Timings for the computationally most demanding steps within the ω-CDD-RI-RPA-NMR calculation for the DNA fragment (DNA)_2_ computed using dense matrix algebra and a linear alkane which was computed using sparse matrix algebra. The cc-pwCVDZ basis set with the corresponding RI basis set was used. Note that the timings for the **B**-field derivative of the self-energy in the positive and negative imaginary time domain are summarized in **Σ**^**B**^(iτ). Calculations were performed on a compute node with 1 TB of RAM without employing batching.

In the following, the timings for the most time-consuming steps within the calculation of ω-CDD-RI-RPA-NMR shieldings are investigated. Please note that the total timings refer to the time for the RPA NMR correlation contribution only, that is, excluding the time for the preceding HF NMR calculation. We computed a DNA fragment with two adenine-thymine base pairs (128 atoms; 580 electrons) using the cc-pwCVDZ basis set (*N* = 1646) with the corresponding RI basis set. Further, as an example of sparse systems, we computed the linear alkane C_60_H_122_ (182 atoms; 482 electrons) using the same basis set (*N* = 1690). No batching was employed for the calculations, which were carried out on a compute node with AMD EPYC 7452 processors with 128 threads, 1 TB of RAM, and 4.7 TB of disk space. The results are shown in Figure 4. For both systems the computation of the **B**-field derivative of the self-energy is the most compute intensive step, followed by the computation of **X**_0_^**B**^(iτ) as well as **Y**^**B**^(iτ). However, for the sparse system, i.e., C_60_H_122_, the timings for all three steps are on the same order while for the dense system, i.e., (DNA)_2_, the computation of **Σ**^**B**^(iτ) is still dominating. This demonstrates that for the linear alkane the sparsity of **Σ**^**B**^(iτ) is efficiently exploited using sparse matrix algebra.

**Figure 5:**
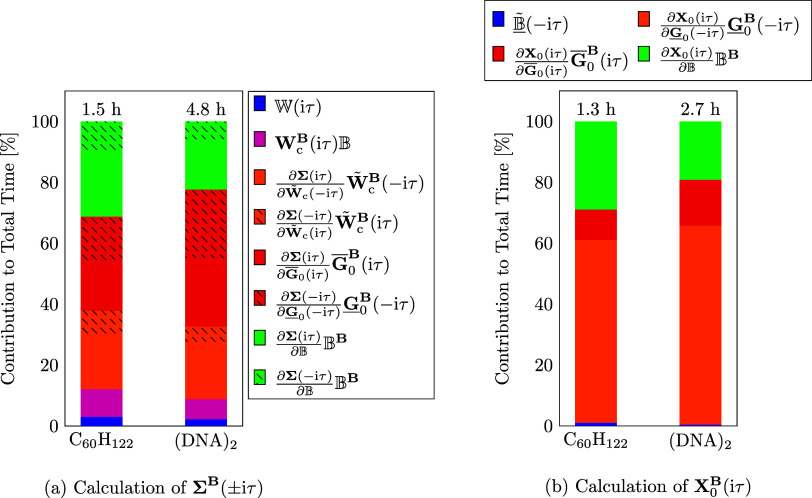
Contribution of the partial derivative terms (a) for the computation of **Σ**^**B**^(iτ) (nonshaded bars) and **Σ**^**B**^(−iτ) (shaded bars) as well as (b) for the computation of **X**_0_^**B**^(iτ) to the total time required for the computation of **Σ**^**B**^(±iτ) and **X**_0_^**B**^(iτ), respectively.

Next, detailed timings for the partial derivatives within the calculation of **Σ**^**B**^(iτ) (∀τ ∈ (−∞, +∞)) and **X**_0_^**B**^(iτ) are investigated. The results are shown in Figure 5. Starting with the timings for **Σ**^**B**^(±iτ) given on the left-hand side of Figure 5, it can be observed that the dominating step is the partial derivative term containing the derivative of the Green’s function. Since CD cannot be applied to this term, it is the most demanding step in terms of computational resources as well as memory requirements. However, for the linear alkane the sparsity introduced through the local metric in the three-center integral tensor is exploited using sparse algebra, which lowers its contribution to the total time compared to the dense DNA fragment. Further, it is evident that the computation of the **B**-field derivative of the self-energy is more efficient for the negative imaginary time domain than for the positive imaginary time domain. This is due to the very efficient CD of the ground state density matrix within the calculation of **Σ**^**B**^(−iτ), while the CD of the Green’s function in the positive imaginary time domain within the calculation of **Σ**^**B**^(iτ) is less effective. For the computation of **X**_0_^**B**^(iτ), shown on the right-hand side of Figure 5, the partial derivative term containing **G**_0_^**B**^(−iτ) requires over 50% of the total time for both systems. For this term only the CD of **G̅**_0_(iτ) can be used, while the more efficient CD of the ground state density matrix can only be used for the remaining terms.

#### 4.3.2 Batching: Linear Alkanes

As described in Section 2.4.1, the introduced batching method was designed to account for the sparsity of intermediates when computing the number of batches. To test this aspect of our batching method, we performed calculations on linear alkanes of increasing length using the cc-pwCVDZ atomic orbital basis set with the corresponding RI basis set. All calculations were performed on a compute note with AMD EPYC 7302 processors using 64 threads, 250 GB of RAM, and 1.7 TB of disk space.

**Figure 6:**
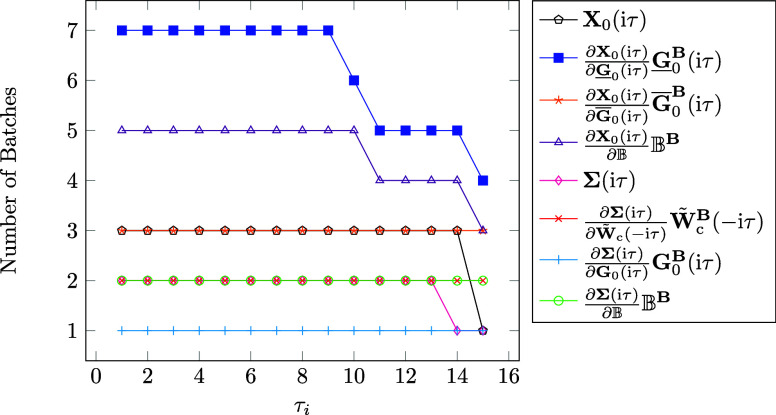
Number of aux-batches for the calculation of the most memory demanding steps per τ quadrature point for the linear alkane C_100_H_202_. Note that τ_*i*_ denotes the *i*th τ quadrature point. Calculations were carried out on a compute node using 250 GB of RAM.

As has been shown in Section 2.4.1, the sparsity of intermediates has a dependence on the τ quadrature point, typically increasing with the τ quadrature points. To account for this, we recompute the number of batches for each τ quadrature point. Since the number of batches is dependent on the memory demands of intermediates it would be expected that the number of batches also decreases with the τ quadrature point. Thus, we start our investigation by considering the number of batches for all intermediates for each τ quadrature point using the linear alkane C_100_H_202_. The results are displayed in Figure 6. It can be observed that the computation of the partial derivative term  requires the highest number of batches, which was expected since this term has the highest memory demand given that CD of the ground state density matrix is not possible for that term. However, the number of batches does decrease significantly with increasing τ points due to the increasing sparsity for which our batching is able to accommodate for. The decrease in batches can be observed for all terms except for , , and . For the first term it is due to the fact that **W̃**_c_^**B**^(iτ) is in general a dense matrix, while for the second term the memory demand is dominated by the memory required for the batched three-center integrals in the AO basis as well as the three-center integrals transformed with **L**, which are independent of τ. For the last term the batched **B**-field derivatives of the three-center integrals are loaded into memory for all three magnetic field directions, whose memory is also independent of τ. To summarize, in general the sparsity of intermediates increases with the τ quadrature points, which our batching is able to account for. As demonstrated, the number of computed batches decreases with increasing τ.

**Figure 7:**
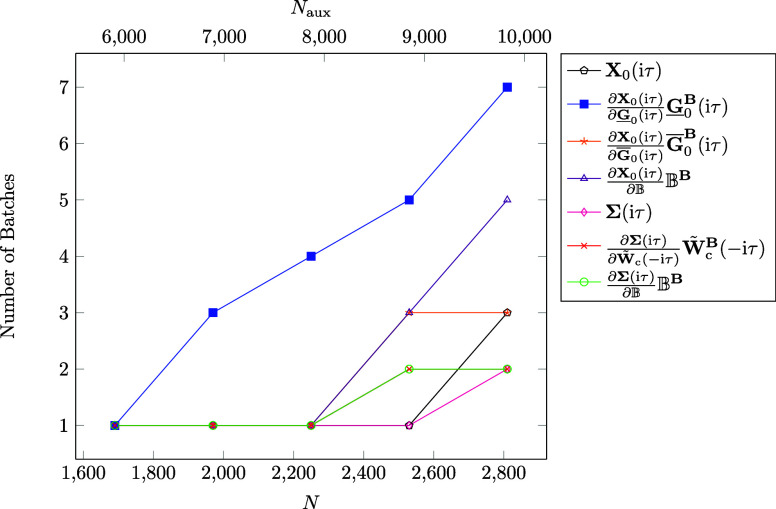
Number of auxiliary function batches for the computation of the partial derivative terms within the calculation of **Σ**^**B**^(iτ) (∀τ ∈ (−∞, +∞)) and **X**_0_^**B**^(iτ) for linear alkanes of increasing size. Note that only the partial derivative terms are displayed, for which the number of batches is larger than 1. Calculations were performed on a compute node with 250 GB of RAM.

Next, we investigate the total number of batches (for the first τ quadrature point) for each intermediate with increasing system sizes. The results are given in Figure 7. Since the number of batches overlap for some intermediates, the results are also summarized in the Supporting Information for clarity. It can be observed again, that the partial derivative term  requires the highest number of batches and increases the strongest with the system size. Further, for the partial derivative terms within the calculation of **Σ**^**B**^(iτ) the number of batches is lower compared to the partial derivative terms for **X**_0_^**B**^(iτ). For the calculation of **Σ**^**B**^(iτ) the batching over auxiliary functions is very beneficial, since various memory demanding intermediates can be computed for one auxiliary function at a time.

**Figure 8:**
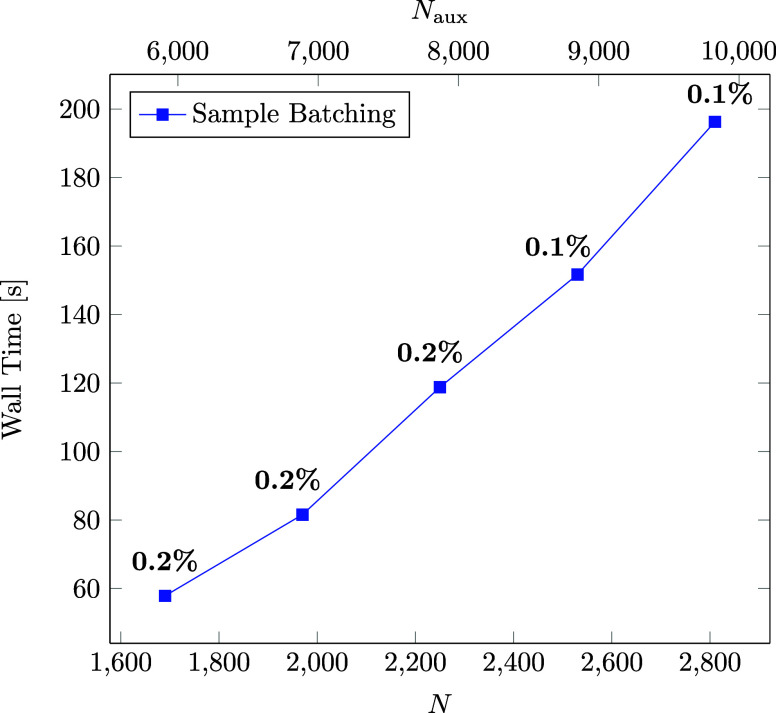
Wall time (s) for the computation of batches using the sample batching method for systems of increasing size with their contribution to the total time for the correlation part of the ω-CDD-RI-RPA-NMR calculation.

For the computation of batches we approximated the memory demands of sparse matrices by sampling the auxiliary function space and precomputing a number of intermediates, which introduces an overhead. To investigate the extend of the overhead, we examine the total time for the computation of batches for increasing system sizes and consider the contribution to the total computation time for the correlation part of the RPA NMR calculation. The results are displayed in Figure 8. The computation time for the sample batching of the largest system size is still under 5 min and the total contribution does not exceed 1% of the total computation time. Therefore, it can be concluded, that the overhead that comes with the sample batching method is practically insignificant.

**Figure 9:**
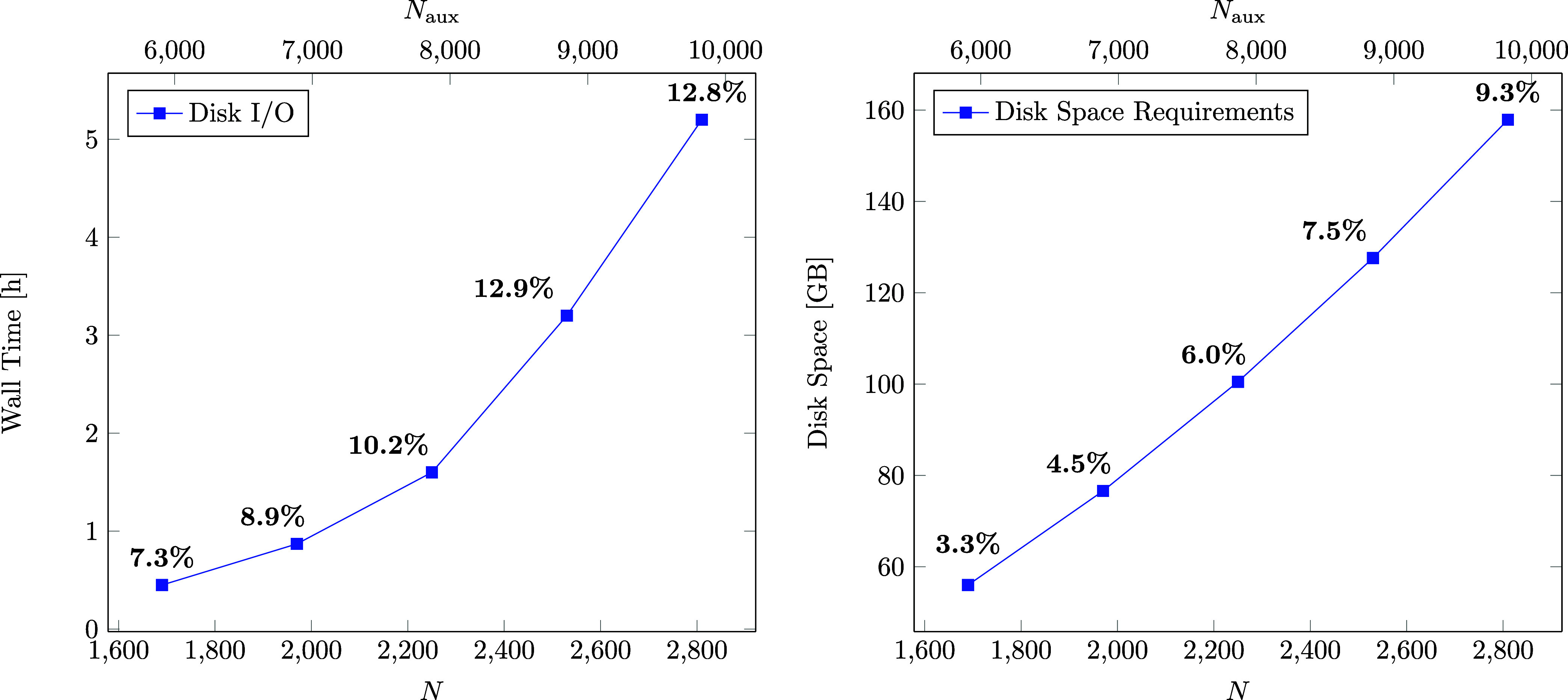
Wall time (h) required for disk I/O operations for increasing system sizes with the contribution to the total time for the correlation part of the ω-CDD-RI-RPA-NMR calculation (left). Further, disk space requirements (GB) for increasing system sizes with the contribution to the total disk space are displayed on the right-hand side.

Another overhead that comes with the batching method is associated with disk input/output (I/O) operations. When using the batching method certain quantities, such as the three-center integrals and their **B**-field derivatives, are stored on disk and read into memory within the respective batching scheme. On the left-hand side of Figure 9 the total time for disk I/O operations as well as the contribution to the total time for the correlation part of the ω-CDD-RI-RPA-NMR calculation is shown for increasing system sizes. As can be seen, the contribution does increase with the system size, due to the increasing number of batches. For the largest system the disk I/O contribution is less than 15% of the total time, which is still acceptable. However, for efficiency reasons an integral direct computation of the three-center integrals and their **B**-field derivatives would certainly be beneficial, but we leave this to future work on this topic.

Lastly, we investigate the maximum disk space requirements of the method for increasing system sizes. The results are summarized on the right-hand side of Figure 9. As can be seen, the disk space demands are low compared to the total disk space, due to the compact storage format we adopted for the three-center integrals and their derivatives as explained in Section 2.4. Therefore, it can be concluded that currently the disk space does not yet constitute a bottleneck in the computation of large system sizes.

In summary, we can conclude this section with the following points:

- The sparsity of intermediates increases with the number of τ quadrature points, which our sparse sample batching is able to account for.
- The time for the computation of batches within the sample batching method is practically insignificant (<1% of the total time).
- The disk space demands are relatively low and currently do not hinder the calculation of large systems.
- Disk I/O operations have a significant contribution to the total time for larger systems, thus, an integral-direct scheme for the three-center integrals and their **B**-field derivatives would be beneficial to explore in future work.

#### 4.3.3 Batching: Illustrative Applications

We further test our batching method on chemically relevant systems representative for potential applications. All calculations were performed on a compute node with AMD EPYC 7452 processors using 128 threads, 1 TB of RAM, and 4.7 TB of disk space.

**Figure 10:**
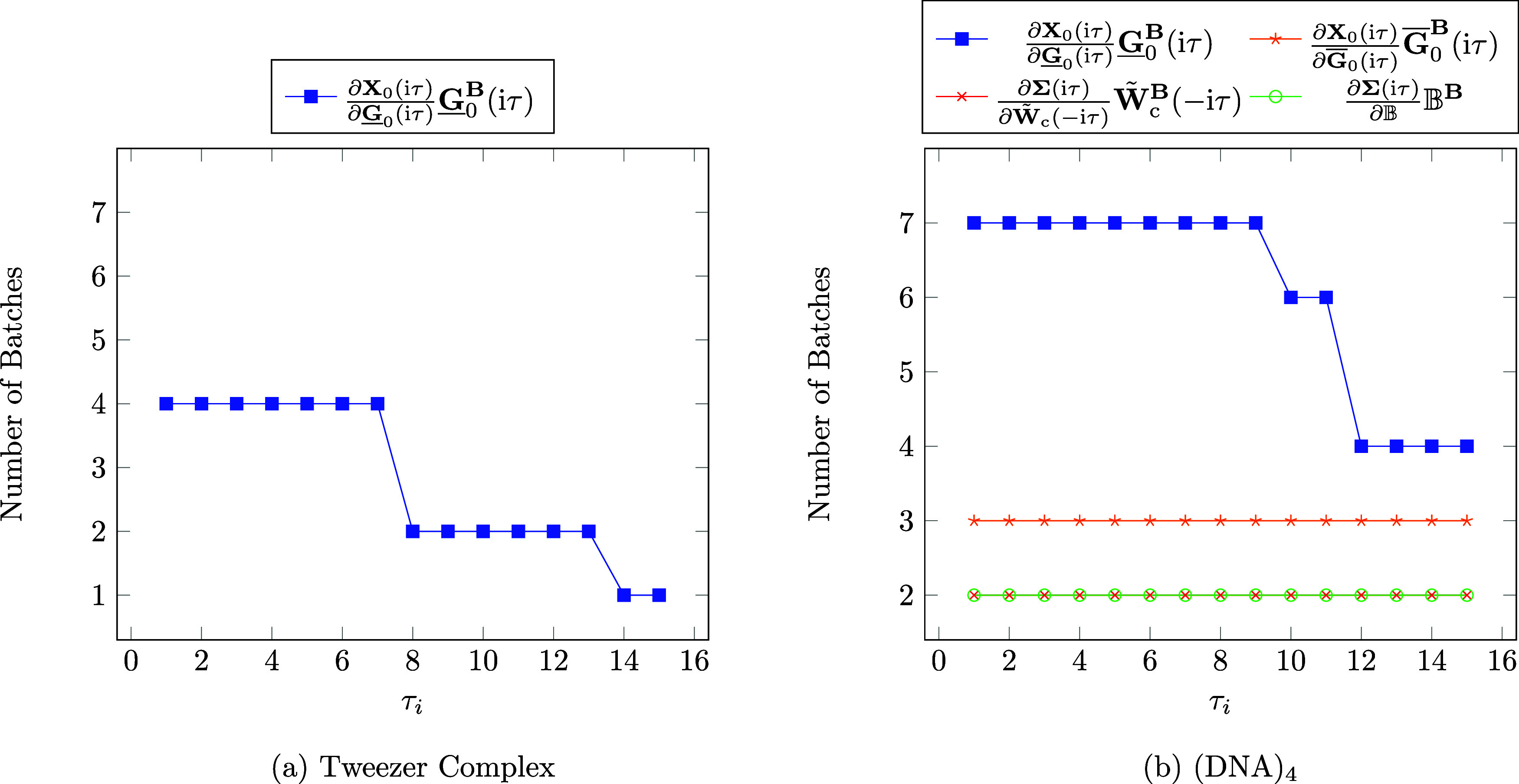
Number of aux-batches for the calculation of intermediates per τ quadrature point for (a) the tweezer complex and (b) (DNA)_4_. Note that τ_*i*_ denotes the *i*th τ quadrature point. Calculations were carried out on a compute node using 1 TB of RAM.

First, we test the performance of our method for a tweezer host–guest complex (92 atoms; 374 electrons) which has been investigated in literature from an application point of view^91^ as well as for performance analysis within NMR calculations.^35,36^ The structure was taken from refs (35,36). The calculation was carried out using a cc-pwCVTZ AO basis set (*N* = 2912) with the corresponding RI basis set. The correlation part of the RPA NMR calculation took 4 days. Here, disk I/O operations accounted for 6.1% of that time and the computation of batches for 0.2%. While disk I/O operations have a significant contribution to the total time, the time for the computation of batches is practically insignificant. The calculation required 541.0 GB of disk space, which is clearly under the available 4.7 TB. Further, on the left-hand side of Figure 10 the number of batches per τ quadrature point is given for all intermediates with a batch number larger than 1. It is interesting to note that for the dense system the number of batches for the intermediate  is also dependent on the τ quadrature point and decreases significantly with increasing τ, which was also observed for sparse systems in the previous section. This shows that our sample batching method is also beneficial for dense systems.

Next, we performed calculations on a DNA strand with four adenine-thymine base pairs, denoted as (DNA)_4_ (260 atoms; 1220 electrons), using a cc-pwCVDZ AO basis set (*N* = 3408) with the corresponding RI basis set. The correlation part of the RPA NMR calculation required 7.5 days in total. Out of that time 5.3% was spend on disk I/O operations, while the sample batching only took 0.2% of the total time. Thus, the time needed for the sample batching method is practically insignificant in this case as well. The time spend on disk I/O operations is not the dominating part, but has still a significant contribution to the total time. The required disk space was 687.3 GB which amount to 14.6% of the available disk space. Next, the number of batches is investigated for all τ quadrature points. The results are given on the right-hand side of Figure 10. Here, only terms are displayed for which the batch count exceeds 1. It can be observed again that the number of batches for the intermediate  shows a strong dependence on the τ quadrature point, making our sample batching relevant for dense systems as well.

## 5 Conclusions

An efficient and low-scaling method for the computation of RPA NMR shielding tensors has been presented that is based on our AO-RI-RPA-NMR method introduced in ref (38). We utilize Cholesky decomposed ground state densities as well as Cholesky decomposed Green’s functions in the positive imaginary time domain. Further, the attenuated Coulomb RI metric was employed to introduce sparsity in the three-center integral tensors, which was efficiently exploited using sparse matrix algebra. Specifically, these techniques were employed for the computation of the response function, self-energy and their **B**-field derivatives which constitute the steepest scaling and most demanding steps in terms of computational effort and memory requirements. It was shown that the introduced approximations do not deteriorate the accuracy of the method. The scaling with the system size was analyzed using linear alkanes, which revealed close to a quadratic scaling.

To lower the memory demand of the method and, thus, extend its applicability to even larger systems, we introduced a batching method for memory demanding intermediates. Here, the memory demand of sparse matrices was approximated by sampling the auxiliary function space and used to compute the number of batches for each τ quadrature point. It was shown that the overhead related to the sampling method has only a small contribution to the total time (<1%) and, thus, is practically insignificant. Further, we analyzed the number of batches for each τ quadrature point for, both, sparse systems and dense systems. We found, that there is a decrease in batches with increasing τ quadrature points for sparse and dense systems. This shows, that our batching method is beneficial for both sparse and dense systems. Further, within the batching method, the three-center integrals and their **B**-field derivatives were stored on disk and read into memory within the respective batching scheme and transformed on the fly. As has been shown, the contribution from disk I/O operations is not the dominating step but it does have a significant contribution to the total time.

In future work, the efficiency of our method could be further improved by utilizing an integral-direct scheme for the integrals and their derivatives and combining the sparse sample batching method with an optimized batching scheme as introduced in ref (54).

The importance of our new NMR method is further highlighted by the possibility to use it as a basis for the implementation of NMR shieldings based on a method that is closely related to RPA, that is σ-functionals.^92,93^ Due to the close relation of σ-functionals and RPA, it should be possible to implement analytical σ-functional NMR shieldings using our efficient RPA NMR implementation. This would provide another accurate and efficient method for the computation of NMR shieldings. Since σ-functionals^92,93^ and extensions thereof^94−96^ are being developed it would be interesting to explore these possibilities in future work.

## Supporting Information Available

Batching algorithms and implementation details for all intermediates; detailed results for the batching of linear alkanes; timings for linear alkanes with dense and sparse matrix algebra employing the ω-CDD-RI-RPA-NMR method, and isotropic NMR shielding constants for (DNA)_4_ (PDF)
