# Supplementary material for: Low-Scaling, Efficient and Memory Optimized Computation of Nuclear Magnetic Resonance Shieldings within the Random Phase Approximation Using Cholesky-Decomposed Densities and an Attenuated Coulomb Metric
Source: J Phys Chem A. 2024 Sep 6;128(37):7950–65. doi: 10.1021/acs.jpca.4c02773 (PMC11421095; doi:10.1021/acs.jpca.4c02773)
Supplement: Supplementary file 1 — jp4c02773_si_001.pdf [file jp4c02773_si_001.pdf]

Supporting Information: Low-Scaling, Efficient and Memory Optimized Computation of Nuclear Magnetic Resonance Shieldings within the Random Phase Approximation using Cholesky-Decomposed Densities and an Attenuated Coulomb Metric

Viktoria Drontschenko[†] and Christian Ochsenfeld[*,†,‡]

†*Chair of Theoretical Chemistry, Department of Chemistry, University of Munich (LMU), D-81377 Munich, Germany*

‡*Max Planck Institute for Solid State Research, D-70569 Stuttgart, Germany*

E-mail: christian.ochsenfeld@cup.uni-muenchen.de

Contents

1 Memory Efficient Implementation **S-3**

1.1 Batching Scheme: $\boldsymbol{\Sigma}(\mathrm{i}\tau)$ S-3

1.2 Batching Scheme: $\mathbf{X}_0(\mathrm{i}\tau)$ S-4

1.3 Batching Scheme: $\boldsymbol{\Sigma}^{\mathbf{B}}(\mathrm{i}\tau)$ S-5

1.3.1 Partial Derivative Term: $\frac{\partial \boldsymbol{\Sigma}(\pm \mathrm{i}\tau)}{\partial \mathbb{B}}\frac{\partial \mathbb{B}}{\partial \mathbf{B}}$ S-6

1.3.2 Partial Derivative Term: $\frac{\partial \boldsymbol{\Sigma}(\pm \mathrm{i}\tau)}{\partial \tilde{\mathbf{W}}_{\mathrm{c}}(\mp \mathrm{i}\tau)}\frac{\partial \tilde{\mathbf{W}}_{\mathrm{c}}(\mp \mathrm{i}\tau)}{\partial \mathbf{B}}$ S-7

1.3.3 Partial Derivative Term: $\frac{\partial \boldsymbol{\Sigma}(\pm \mathrm{i}\tau)}{\partial \mathbf{G}_0(\pm \mathrm{i}\tau)}\frac{\partial \mathbf{G}_0(\pm \mathrm{i}\tau)}{\partial \mathbf{B}}$ S-8

1.4 Batching Scheme: $\mathbf{X}_0^{\mathbf{B}}(\mathrm{i}\tau)$ S-9

1.4.1 Partial Derivative Term: $\frac{\partial \mathbf{X}_0(\mathrm{i}\tau)}{\partial \mathbb{B}}\frac{\partial \mathbb{B}}{\partial \mathbf{B}}$ S-9

1.4.2 Partial Derivative Term: $\frac{\partial \mathbf{X}_0(\mathrm{i}\tau)}{\partial \underline{\mathbf{G}}_0(-\mathrm{i}\tau)}\frac{\partial \underline{\mathbf{G}}_0(-\mathrm{i}\tau)}{\partial \mathbf{B}}$ S-10

1.4.3 Partial Derivative Term: $\frac{\partial \mathbf{X}_0(\mathrm{i}\tau)}{\partial \overline{\mathbf{G}}_0(\mathrm{i}\tau)}\frac{\partial \overline{\mathbf{G}}_0(\mathrm{i}\tau)}{\partial \mathbf{B}}$ S-11

2 Batching-Results: Linear Alkanes **S-12**

3 Timings: Dense vs. Sparse Implementation **S-13**

4 Isotropic NMR shielding constants for $(\mathrm{DNA})_4$/cc-pwCVDZ **S-13**

1 Memory Efficient Implementation

1.1 Batching Scheme: $\mathbf{\Sigma}(\mathrm{i}\tau)$

The computation of the self-energy is shown in Algorithm 1. First the intermediate $\mathbb{W}$ is computed (lines 1-12) and written on disk. For efficiency, lines 5-9 are implemented as a matrix multiplication. Then, within a separate batching scheme, $\mathbb{W}$ and the three-center integrals are read into memory for one auxiliary function batch (aux-batch) (lines 14 and 15, respectively). It should be noted that quantities are read into memory for one aux-batch at a time, rather than one auxiliary function, in order to optimize reading performance, since processing larger data in fewer instances is more efficient than repeatedly processing smaller amounts of data. Then, in lines 17-21, the self-energy in the negative imaginary time domain is computed for one auxiliary function at a time. In lines 23-27 the self-energy in the positive imaginary time domain is computed, again, for one auxiliary function at a time. To increase computational efficiency, we parallelize over the auxiliary function indices (lines 17-21 and 23-27). It should be noted that $\mathbf{\Sigma}(\mathrm{i}\tau)$ and $\mathbf{\Sigma}(-\mathrm{i}\tau)$ are computed within the same batching scheme, since the memory demanding third order tensors are computed for one auxiliary function at a time and, thus, do not show high memory requirements. The memory requirements for the batching method are rather determined by the memory demands of $\mathbb{W}$ and $\mathbb{B}$, which are loaded into memory for one aux-batch.

Algorithm 1 $\mathbf{\Sigma}(\pm \mathrm{i}\tau)$ per $\tau \geq 0$

▷ Calculate $\mathbb{W}^P(\mathrm{i}\tau)(\forall P)$ and write on disk
for aux-batch1 **do**
  for aux-batch2 **do**
    read $\mathbf{B}^Q \quad \forall Q \in$ aux-batch2
    for $P \in$ aux-batch1 **do**
      for $Q \in$ aux-batch2 **do**
        $\mathbb{W}^P_{\mu\nu}(\mathrm{i}\tau) \mathrel{+}= W_{\mathrm{c},PQ}(\mathrm{i}\tau)\mathbb{B}^Q_{\mu\nu} \quad \forall \mu, \nu$
      end for
    end for
  end for
  write on disk $\mathbb{W}^P_{\mu\nu}(\mathrm{i}\tau) \quad \forall \mu, \nu, P \in$ aux-batch1
end for
for aux-batch1 **do**
  read $\mathbb{W}^P(\mathrm{i}\tau) \quad \forall P \in$ aux-batch1
  read $\mathbb{B}^P \quad \forall P \in$ aux-batch1
  ▷ Calculate $\mathbf{\Sigma}(-\mathrm{i}\tau)$
  for $P \in$ aux-batch1 **do** ▷ parallel
    $\underline{\mathbb{W}}^P_{\mu\underline{i}}(\mathrm{i}\tau) = \mathbb{W}^P_{\mu\nu}(\mathrm{i}\tau) L_{\nu\underline{i}} \quad \forall \mu, \underline{i}$
    $\underline{\mathbb{B}}^P_{\nu\underline{j}} = \mathbb{B}^P_{\nu\lambda} L_{\lambda\underline{j}} \quad \forall \nu, \underline{j}$
    $\Sigma_{\mu\nu}(-\mathrm{i}\tau) \mathrel{+}= \underline{\mathbb{W}}^P_{\mu\underline{i}}(\mathrm{i}\tau) \underline{\underline{G}}_{0,\underline{i}\underline{j}}(-\mathrm{i}\tau) \underline{\mathbb{B}}^P_{\nu\underline{j}} \quad \forall \mu, \nu$
  end for
  ▷ Calculate $\mathbf{\Sigma}(\mathrm{i}\tau)$
  for $P \in$ aux-batch1 **do** ▷ parallel
    $\overline{\mathbb{W}}^P_{\mu\underline{a}}(\mathrm{i}\tau) = \mathbb{W}^P_{\mu\nu}(\mathrm{i}\tau) L_{\mathrm{virt},\nu\underline{a}}(\mathrm{i}\tau) \quad \forall \mu, \underline{a}$
    $\overline{\mathbb{B}}^P_{\nu\underline{a}}(\mathrm{i}\tau) = \mathbb{B}^P_{\nu\lambda} L_{\mathrm{virt},\lambda\underline{a}}(\mathrm{i}\tau) \quad \forall \nu, \underline{a}$
    $\Sigma_{\mu\nu}(\mathrm{i}\tau) \mathrel{+}= \overline{\mathbb{W}}^P_{\mu\underline{a}}(\mathrm{i}\tau) \overline{\mathbb{B}}^P_{\nu\underline{a}}(\mathrm{i}\tau) \quad \forall \mu, \nu$
  end for
end for

1.2 Batching Scheme: $\mathbf{X}_0(\mathrm{i}\tau)$

For the computation of the response function in Algorithm 2, the three-center integrals are read into memory for one aux-batch (line 2) and subsequently transformed with the Cholesky matrix $\mathbf{L}$ (line 4) and used for the computation of the intermediate $\tilde{\mathbb{B}}^P_{\underline{ai}}(\mathrm{i}\tau)$ (line 5). In a second aux-batch loop, the three-center integrals are read into memory for the respective aux-batch (line 8) and used to compute the intermediate $\overline{\underline{\mathbb{B}}}^Q_{\underline{ai}}(\mathrm{i}\tau)$. Finally, in line 14 the response function is computed by multiplying both intermediates. It should be noted that the symmetry of the response function is exploited for the second aux-batch loop (lines 7-17). Further, lines 12-16 are implemented as a matrix multiplication. The loops over the auxiliary function indices are parallelized (lines 3-6 and 9-11).

Algorithm 2 $\mathbf{X}_0(\mathrm{i}\tau)$ per $\tau \geq 0$

for aux-batch1 **do**
  read $\mathbb{B}^P_{\mu\nu} \quad \forall \mu, \nu, P \in$ aux-batch1
  for $P \in$ aux-batch1 **do** ▷ parallel
    $\mathbb{B}^P_{\mu\underline{j}} = \mathbb{B}^P_{\mu\nu} L_{\nu\underline{j}} \quad \forall \underline{j}, \mu$
    $\tilde{B}^P_{\underline{a}\underline{i}}(\mathrm{i}\tau) = \underline{\underline{G}}_{0,\underline{i}\underline{j}}(-\mathrm{i}\tau)\mathbb{B}^P_{\mu\underline{j}} L_{\mathrm{virt},\mu\underline{a}}(\mathrm{i}\tau) \quad \forall \underline{i}, \underline{a}$
  end for
  for aux-batch2 $\geq$ aux-batch1 **do**
    read $\mathbb{B}^Q_{\mu\nu} \quad \forall \mu, \nu, Q \in$ aux-batch2
    for $Q \in$ aux-batch2 **do** ▷ parallel
      $\overline{\mathbb{B}}^Q_{\underline{a}\underline{i}}(\mathrm{i}\tau) = L_{\mathrm{virt},\mu\underline{a}}(\mathrm{i}\tau)\mathbb{B}^Q_{\mu\nu} L_{\nu\underline{i}} \quad \forall \underline{i}, \underline{a}$
    end for
    for $P \in$ aux-batch1 **do**
      for $Q \in$ aux-batch2 **do**
        $X_{0,PQ}(\mathrm{i}\tau) = \tilde{B}^P_{\underline{a}\underline{i}}(\mathrm{i}\tau)\overline{\mathbb{B}}^Q_{\underline{a}\underline{i}}(\mathrm{i}\tau)$
      end for
    end for
  end for
end for

1.3 Batching Scheme: $\mathbf{\Sigma}^{\mathbf{B}}(\mathrm{i}\tau)$

A general outline for the computation of $\mathbf{\Sigma}^{\mathbf{B}}(\mathrm{i}\tau)$ is provided in Algorithm 3. First, the partial derivative with respect to $\tilde{\mathbf{W}}_{\mathrm{c}}(-\mathrm{i}\tau)$ is computed (line 2). Next, the intermediate $\mathbb{W}(\mathrm{i}\tau)$ is computed and written on disk as shown in Algorithm 1 lines 1-13. This intermediate is read into memory for the computation of the remaining partial derivatives, that is, the partial derivative with respect to the Green's function (line 4) and three-center integrals (line 5). All partial derivative terms (lines 2-4) are computed for the **B**-field derivative of the self-energy in the positive and negative imaginary time domain within the same batching scheme.

Algorithm 3 $\mathbf{\Sigma}^{\mathbf{B}}(\mathrm{i}\tau) \quad \forall \tau \in (-\infty, +\infty)$

1: **for all** τ **do**
2:   calc. $\frac{\partial \mathbf{\Sigma}(\mathrm{i}\tau)}{\partial \tilde{\mathbf{W}}_{\mathrm{c}}(-\mathrm{i}\tau)} \frac{\partial \tilde{\mathbf{W}}_{\mathrm{c}}(-\mathrm{i}\tau)}{\partial \mathbf{B}}$
3:   calc. batched $\mathbb{W}^P(\mathrm{i}\tau)(\forall P)$; write on disk
4:   calc. $\frac{\partial \mathbf{\Sigma}(\mathrm{i}\tau)}{\partial \mathbf{G}_0(\mathrm{i}\tau)} \frac{\partial \mathbf{G}_0(\mathrm{i}\tau)}{\partial \mathbf{B}}$
5:   calc. $\frac{\partial \mathbf{\Sigma}(\mathrm{i}\tau)}{\partial \mathbb{B}^P} \frac{\partial \mathbb{B}^P}{\partial \mathbf{B}}(\forall P)$
6: **end for**

Algorithm 4 $\mathbf{X}^{\mathbf{B}}_0(\mathrm{i}\tau) \quad \forall \tau \geq 0$

for all τ **do**
  calc. $\frac{\partial \mathbf{X}_0(\mathrm{i}\tau)}{\partial \mathbf{G}_0(-\mathrm{i}\tau)} \frac{\partial \mathbf{G}_0(-\mathrm{i}\tau)}{\partial \mathbf{B}}$
  calc. batched $\tilde{\underline{\mathbb{B}}}^P_{\mu\underline{i}}(-\mathrm{i}\tau)(\forall P, \mu, \underline{i})$; write on disk
  calc. $\frac{\partial \mathbf{X}_0(\mathrm{i}\tau)}{\partial \mathbf{G}_0(\mathrm{i}\tau)} \frac{\partial \mathbf{G}_0(\mathrm{i}\tau)}{\partial \mathbf{B}}$
  calc. $\frac{\partial \mathbf{X}_0(\mathrm{i}\tau)}{\partial \mathbb{B}^P} \frac{\partial \mathbb{B}^P}{\partial \mathbf{B}}(\forall P)$
end for

1.3.1 Partial Derivative Term: $\frac{\partial \mathbf{\Sigma}(\pm \mathrm{i}\tau)}{\partial \mathbb{B}} \frac{\partial \mathbb{B}}{\partial \mathbf{B}}$

The computation of the partial derivative with respect to the three-center integrals is shown Algorithm 5. Here, first, $\mathbb{W}(\mathrm{i}\tau)$ is read into memory for one aux-batch. Next, the **B**-field derivatives of the three-center integrals are read into memory for one aux-batch and all magnetic field directions. Next, the contribution to $\mathbf{\Sigma}^{\mathbf{B}}(-\mathrm{i}\tau)$ is computed for one aux-function at a time (lines 7-13). Here, the intermediate $\underline{\mathbb{W}}(\mathrm{i}\tau)$ is computed first and subsequently used to compute the contribution to $\mathbf{\Sigma}^{\mathbf{B}}(-\mathrm{i}\tau)$ for one magnetic field direction at a time. The contribution to $\mathbf{\Sigma}^{\mathbf{B}}(\mathrm{i}\tau)$ is computed in lines 15-21. A similar loop structure is used, starting with a loop over auxiliary functions, where $\overline{\mathbb{W}}(\mathrm{i}\tau)$ is computed and used to compute the contribution to $\mathbf{\Sigma}^{\mathbf{B}}(\mathrm{i}\tau)$ (lines 18 and 19) for one **B**-field direction at a time. The loops over the auxiliary function indices (lines 7-13 and 15-21) are parallelized. Since the contribution to the derivative of the self-energy in the positive and negative imaginary time domain is computed for one auxiliary function at a time, the memory demand of this algorithm is determined by $\mathbb{W}(\mathrm{i}\tau)$ and the derivatives of the three-center integrals. Therefore, it is sensible to combine the computation of the partial derivatives of the self-energy in the positive and negative imaginary time domain in one batching scheme.

Algorithm 5 $\frac{\partial \boldsymbol{\Sigma}(\pm \mathrm{i}\tau)}{\partial \mathbb{B}} \frac{\partial \mathbb{B}}{\partial \mathbf{B}}$ per $\tau \geq 0$

for aux-batch1 **do**
    read $\mathbb{W}^P(\mathrm{i}\tau) \quad \forall P \in$ aux-batch1
    for $B \in \mathbf{B} = \{B_x, B_y, B_z\}$ **do**
        read $\left(\mathbb{B}^P_{\lambda\nu}\right)^B \quad \forall \lambda, \nu, P \in$ aux-batch1
    end for
    ▷ Calculate contribution to $\boldsymbol{\Sigma}^{\mathbf{B}}(-\mathrm{i}\tau)$
    for $P \in$ aux-batch1 **do** ▷ parallel
        $\underline{\mathbb{W}}^P_{\mu\underline{j}}(\mathrm{i}\tau) = \mathbb{W}^P_{\mu\nu}(\mathrm{i}\tau) L_{\nu\underline{i}} \underline{\underline{G}}_{0,\underline{ij}}(-\mathrm{i}\tau) \quad \forall \mu, \underline{j}$
        for $B \in \mathbf{B} = \{B_x, B_y, B_z\}$ **do**
            $\Sigma^B_{\mu\nu}(-\mathrm{i}\tau) \mathrel{+}= \underline{\mathbb{W}}^P_{\mu\underline{j}}(\mathrm{i}\tau) \left(\mathbb{B}^P_{\lambda\nu}\right)^B L_{\lambda\underline{j}} \quad \forall \mu, \nu$
            $\Sigma^B_{\nu\mu}(-\mathrm{i}\tau) \mathrel{-}= \Sigma^B_{\mu\nu}(-\mathrm{i}\tau) \quad \forall \mu, \nu$
        end for
    end for
    ▷ Calculate contribution to $\boldsymbol{\Sigma}^{\mathbf{B}}(\mathrm{i}\tau)$
    for $P \in$ aux-batch1 **do** ▷ parallel
        $\overline{\overline{\mathbb{W}}}^P_{\mu\underline{a}}(\mathrm{i}\tau) = \mathbb{W}^P_{\mu\nu}(\mathrm{i}\tau) L_{\mathrm{virt},\nu\underline{a}}(\mathrm{i}\tau) \quad \forall \mu, \underline{a}$
        for $B \in \mathbf{B} = \{B_x, B_y, B_z\}$ **do**
            $\Sigma^B_{\mu\nu}(\mathrm{i}\tau) \mathrel{+}= \overline{\overline{\mathbb{W}}}^P_{\mu\underline{a}}(\mathrm{i}\tau) \left(\mathbb{B}^P_{\lambda\nu}\right)^B L_{\mathrm{virt},\lambda\underline{a}}(\mathrm{i}\tau) \quad \forall \mu, \nu$
            $\Sigma^B_{\nu\mu}(\mathrm{i}\tau) \mathrel{-}= \Sigma^B_{\mu\nu}(\mathrm{i}\tau) \quad \forall \mu, \nu$
        end for
    end for
end for

1.3.2 Partial Derivative Term: $\frac{\partial \boldsymbol{\Sigma}(\pm \mathrm{i}\tau)}{\partial \tilde{\mathbf{W}}_{\mathbf{c}}(\mp \mathrm{i}\tau)} \frac{\partial \tilde{\mathbf{W}}_{\mathbf{c}}(\mp \mathrm{i}\tau)}{\partial \mathbf{B}}$

Algorithm 6 displays the detailed procedure for the computation of the partial derivative $\frac{\partial \boldsymbol{\Sigma}(\pm \mathrm{i}\tau)}{\partial \mathbf{W}_{\mathrm{c}}(\mp \mathrm{i}\tau)} \frac{\partial \mathbf{W}_{\mathrm{c}}(\mp \mathrm{i}\tau)}{\partial \mathbf{B}}$. The contributions to $\boldsymbol{\Sigma}^{\mathbf{B}}(-\mathrm{i}\tau)$ and $\boldsymbol{\Sigma}^{\mathbf{B}}(\mathrm{i}\tau)$ are computed within the same batching scheme. Lines 5-9 are implemented as a matrix multiplication.

Algorithm 6 $\frac{\partial \boldsymbol{\Sigma}(\pm i\tau)}{\partial \tilde{\mathbf{W}}_c(\mp i\tau)} \frac{\partial \tilde{\mathbf{W}}_c(\mp i\tau)}{\partial \mathbf{B}}$ per $\tau \geq 0$

for aux-batch1 **do**
  for aux-batch2 **do**
    read $\mathbb{B}^Q \quad \forall Q \in$ aux-batch2
    for $B \in \mathbf{B} = \{B_x, B_y, B_z\}$ **do**
      for $P \in$ aux-batch1 **do**
        for $Q \in$ aux-batch2 **do**
          $\left(\mathbb{W}^P_{\mu\nu}\right)^B(i\tau) = W^B_{c,PQ}(i\tau)\mathbb{B}^Q_{\mu\nu} \quad \forall \mu, \nu$
        end for
      end for
    end for
  end for
  read $\mathbb{B}^P \quad \forall P \in$ aux-batch1
  ▷ Calculate contribution to $\boldsymbol{\Sigma}^{\mathbf{B}}(-i\tau)$
  for $P \in$ aux-batch1 **do** ▷ parallel
    $\underline{\mathbb{B}}^P_{\nu\underline{j}} = \mathbb{B}^P_{\nu\mu} L_{\mu\underline{j}} \quad \forall \nu, \underline{j}$
    $\tilde{\underline{\mathbb{B}}}^P_{\nu\underline{i}}(-i\tau) = \underline{\underline{G}}_{0,\underline{ij}}(-i\tau)\underline{\mathbb{B}}^P_{\nu\underline{j}} \quad \forall \nu, \underline{i}$
    for $B \in \mathbf{B} = \{B_x, B_y, B_z\}$ **do**
      $\left(\tilde{\mathbb{W}}^P_{\mu\underline{i}}\right)^B(i\tau) = \left(\mathbb{W}^P_{\mu\nu}\right)^B(i\tau) L_{\nu\underline{i}} \quad \forall \mu, \nu, \underline{i}$
      $\Sigma^B_{\mu\nu}(-i\tau) \mathrel{+}= \left(\tilde{\mathbb{W}}^P_{\mu\underline{i}}\right)^B(i\tau)\tilde{\underline{\mathbb{B}}}^P_{\nu\underline{i}}(-i\tau) \quad \forall \mu, \nu$
    end for
  end for
  ▷ Calculate contribution to $\boldsymbol{\Sigma}^{\mathbf{B}}(i\tau)$
  for $P \in$ aux-batch1 **do** ▷ parallel
    $\overline{\mathbb{B}}^P_{\mu\underline{a}} = \mathbb{B}^P_{\mu\nu} L_{\mathrm{virt},\nu\underline{a}}(i\tau) \quad \forall \mu, \underline{a}$
    for $B \in \mathbf{B} = \{B_x, B_y, B_z\}$ **do**
      $\left(\tilde{\mathbb{W}}^P_{\mu\underline{a}}\right)^B(i\tau) = \left(\mathbb{W}^P_{\mu\nu}\right)^B(i\tau) L_{\mathrm{virt},\nu\underline{a}}(i\tau) \quad \forall \mu, \underline{a}$
      $\Sigma^B_{\mu\nu}(i\tau) \mathrel{+}= \left(\tilde{\mathbb{W}}^P_{\mu\underline{a}}\right)^B(i\tau)\overline{\mathbb{B}}^P_{\nu\underline{a}}(i\tau) \quad \forall \mu, \nu$
    end for
  end for
end for

1.3.3 Partial Derivative Term: $\frac{\partial \boldsymbol{\Sigma}(\pm i\tau)}{\partial \mathbf{G}_0(\pm i\tau)} \frac{\partial \mathbf{G}_0(\pm i\tau)}{\partial \mathbf{B}}$

Algorithm 7 shows the computation of the partial derivative $\frac{\partial \boldsymbol{\Sigma}(\pm i\tau)}{\partial \mathbf{G}_0(\pm i\tau)} \frac{\partial \mathbf{G}_0(\pm i\tau)}{\partial \mathbf{B}}$. The contributions to $\boldsymbol{\Sigma}^{\mathbf{B}}(-i\tau)$ and $\boldsymbol{\Sigma}^{\mathbf{B}}(i\tau)$ are computed within the same batching scheme.

Algorithm 7 $\frac{\partial \mathbf{\Sigma}(\pm \mathrm{i}\tau)}{\partial \mathbf{G}_0(\pm \mathrm{i}\tau)} \frac{\partial \mathbf{G}_0(\pm \mathrm{i}\tau)}{\partial \mathbf{B}}$ per $\tau \geq 0$

for aux-batch1 **do**
  read $\mathbb{B}^P \quad \forall P \in$ aux-batch1
  read $\mathbb{W}^P(\mathrm{i}\tau) \quad \forall P \in$ aux-batch1
  ▷ Calculate contribution to $\mathbf{\Sigma}^{\mathbf{B}}(-\mathrm{i}\tau)$
  for $P \in$ aux-batch1 **do** ▷ parallel
    for $B \in \mathbf{B} = \{B_x, B_y, B_z\}$ **do**
      $\Sigma^B_{\mu\nu}(-\mathrm{i}\tau) \mathrel{-}= \mathbb{W}^P_{\mu\lambda} \underline{G}^B_{0,\lambda\sigma}(-\mathrm{i}\tau) \mathbb{B}^P_{\sigma\nu} \quad \forall \mu, \nu$
    end for
  end for
  ▷ Calculate contribution to $\mathbf{\Sigma}^{\mathbf{B}}(\mathrm{i}\tau)$
  for $P \in$ aux-batch1 **do** ▷ parallel
    for $B \in \mathbf{B} = \{B_x, B_y, B_z\}$ **do**
      $\Sigma^B_{\mu\nu}(\mathrm{i}\tau) \mathrel{-}= \mathbb{W}^P_{\mu\lambda} \overline{G}^B_{0,\lambda\sigma}(\mathrm{i}\tau) \mathbb{B}^P_{\sigma\nu} \quad \forall \mu, \nu$
    end for
  end for
end for

1.4 Batching Scheme: $\mathbf{X}_0^{\mathbf{B}}(\mathrm{i}\tau)$

A general outline for the computation of $\mathbf{X}_0^{\mathbf{B}}(\mathrm{i}\tau)$ is provided in Algorithm 4.

$$\underline{\underline{\tilde{\mathbb{B}}}}^P_{\mu\underline{i}}(-\mathrm{i}\tau) := \underline{\underline{G}}_{0,\underline{i}\underline{j}}(-\mathrm{i}\tau) \underline{\underline{\mathbb{B}}}^P_{\mu\underline{j}} \tag{1}$$

is precomputed and written on disk (line 3) by batching over the auxiliary function index. This intermediate is then read into memory for the computation of the partial derivative with respect to $\overline{\mathbf{G}}_0(\mathrm{i}\tau)$ (line 4) as well as $\mathbb{B}$ (line 5).

1.4.1 Partial Derivative Term: $\frac{\partial \mathbf{X}_0(\mathrm{i}\tau)}{\partial \mathbb{B}} \frac{\partial \mathbb{B}}{\partial \mathbf{B}}$

The detailed computation of the partial derivative with respect to the three-center integrals is shown in Algorithm 8. First, the intermediate $\underline{\underline{\tilde{\mathbb{B}}}}(-\mathrm{i}\tau)$ is read into memory for one aux-batch (line 2) and transformed with $\mathbf{L}_{\mathrm{virt}}(\mathrm{i}\tau)$ (line 4). In a second aux-batch loop, the **B**-field derivatives of the three-center integrals are read into memory for one **B**-field direction and one aux-batch and subsequently transformed in line 10. Finally, in lines 12-17 the derivative of the response function is computed per **B**-field direction. Lines 12-17 are implemented as a matrix multiplication. Further, the loops over the auxiliary function indices are parallelized

(lines 3-5 and 9-11).

Algorithm 8 $\frac{\partial \mathbf{X}_0(\mathrm{i}\tau)}{\partial \mathbb{B}} \frac{\partial \mathbb{B}}{\partial \mathbf{B}}$ per $\tau \geq 0$

1: **for** aux-batch1 **do**
2: read $\tilde{\underline{\mathbb{B}}}^P_{\mu\underline{i}}(-\mathrm{i}\tau) \quad \forall \underline{i}, \mu, P \in$ aux-batch1
3: **for** $P \in$ aux-batch1 **do**   ▷ parallel
4:   $\tilde{B}^P_{\underline{ai}}(\mathrm{i}\tau) = \tilde{\underline{\mathbb{B}}}^P_{\mu\underline{i}}(-\mathrm{i}\tau) L_{\mathrm{virt},\mu\underline{a}}(\mathrm{i}\tau) \quad \forall \underline{i}, \underline{a}$
5: **end for**
6: **for** aux-batch2 **do**
7:   **for** $B \in \mathbf{B} = \{B_x, B_y, B_z\}$ **do**
8:    read $\left(\mathbb{B}^Q_{\mu\nu}\right)^B \quad \forall \mu, \nu, Q \in$ aux-batch2
9:    **for** $Q \in$ aux-batch2 **do**   ▷ parallel
10:     $\left(\overline{\mathbb{B}}^Q_{\underline{ai}}\right)^B(\mathrm{i}\tau) = L_{\mathrm{virt},\mu\underline{a}}(\mathrm{i}\tau) \left(\mathbb{B}^Q_{\mu\nu}\right)^B L_{\nu\underline{i}} \quad \forall \underline{i}, \underline{a}$
11:    **end for**
12:    **for** $P \in$ aux-batch1 **do**
13:     **for** $Q \in$ aux-batch2 **do**
14:      $X^B_{0,PQ}(\mathrm{i}\tau) \mathrel{+}= \tilde{B}^P_{\underline{ai}}(\mathrm{i}\tau) \left(\overline{\mathbb{B}}^Q_{\underline{ai}}\right)^B(\mathrm{i}\tau)$
15:      $X^B_{0,QP}(\mathrm{i}\tau) \mathrel{-}= X^B_{0,PQ}(\mathrm{i}\tau)$
16:     **end for**
17:    **end for**
18:   **end for**
19: **end for**
20: **end for**

1.4.2 Partial Derivative Term: $\frac{\partial \mathbf{X}_0(\mathrm{i}\tau)}{\partial \underline{\mathbf{G}}_0(-\mathrm{i}\tau)} \frac{\partial \underline{\mathbf{G}}_0(-\mathrm{i}\tau)}{\partial \mathbf{B}}$

The computation of the partial derivative $\frac{\partial \mathbf{X}_0(\mathrm{i}\tau)}{\partial \underline{\mathbf{G}}_0(-\mathrm{i}\tau)} \frac{\partial \underline{\mathbf{G}}_0(-\mathrm{i}\tau)}{\partial \mathbf{B}}$ is given in Algorithm 9. Lines 15-19 are implemented as a matrix multiplication.

Algorithm 9 $\frac{\partial \mathbf{X}_0(\mathrm{i}\tau)}{\partial \underline{\mathbf{G}}_0(-\mathrm{i}\tau)} \frac{\partial \underline{\mathbf{G}}_0(-\mathrm{i}\tau)}{\partial \mathbf{B}}$ per $\tau \geq 0$

for aux-batch1 **do**
  read $\mathbb{B}^P \quad \forall P \in$ aux-batch1
  for $P \in$ aux-batch1 **do** ▷ parallel
    $\overline{\mathbb{B}}^P_{\mu\underline{a}} = \mathbb{B}^P_{\mu\nu} L_{\mathrm{virt},\nu\underline{a}}(\mathrm{i}\tau) \quad \forall \mu, \underline{a}$
  end for
  for aux-batch2 $\geq$ aux-batch1 **do**
    read $\mathbb{B}^Q \quad \forall Q \in$ aux-batch2
    for $Q \in$ aux-batch2 **do** ▷ parallel
      $\overline{\mathbb{B}}^Q_{\mu\underline{a}}(\mathrm{i}\tau) = \mathbb{B}^Q_{\mu\nu} L_{\mathrm{virt},\nu\underline{a}}(\mathrm{i}\tau) \quad \forall \mu, \underline{a}$
    end for
    for $B \in \mathbf{B} = \{B_x, B_y, B_z\}$ **do**
      for $Q \in$ aux-batch2 **do** ▷ parallel
        $\left(\tilde{\mathbb{B}}^Q_{\mu\underline{a}}\right)^B(\mathrm{i}\tau) = \overline{\mathbb{B}}^Q_{\nu\underline{a}} \underline{G}^B_{0,\nu\mu}(-\mathrm{i}\tau) \quad \forall \mu, \underline{a}$
      end for
      for $P \in$ aux-batch1 **do**
        for $Q \in$ aux-batch2 **do**
          $X^B_{0,PQ}(\mathrm{i}\tau) \mathrel{+}= \overline{\mathbb{B}}^P_{\mu\underline{a}} \left(\tilde{\mathbb{B}}^Q_{\mu\underline{a}}\right)^B$
        end for
      end for
    end for
  end for
end for

1.4.3 Partial Derivative Term: $\frac{\partial \mathbf{X}_0(\mathrm{i}\tau)}{\partial \overline{\mathbf{G}}_0(\mathrm{i}\tau)} \frac{\partial \overline{\mathbf{G}}_0(\mathrm{i}\tau)}{\partial \mathbf{B}}$

The computation of the partial derivative $\frac{\partial \mathbf{X}_0(\mathrm{i}\tau)}{\partial \overline{\mathbf{G}}_0(\mathrm{i}\tau)} \frac{\partial \overline{\mathbf{G}}_0(\mathrm{i}\tau)}{\partial \mathbf{B}}$ is shown in Algorithm 4. Lines 12-16 are implemented as a matrix multiplication.

Algorithm 10 $\frac{\partial \mathbf{X}_0(\mathrm{i}\tau)}{\partial \overline{\mathbf{G}}_0(\mathrm{i}\tau)}\frac{\partial \overline{\mathbf{G}}_0(\mathrm{i}\tau)}{\partial \mathbf{B}}$ per $\tau \geq 0$

for aux-batch1 **do**
    read $\mathbb{B}^P \quad \forall P \in$ aux-batch1
    for $P \in$ aux-batch1 **do** ▷ parallel
        $\underline{\mathbb{B}}^P_{\mu\underline{i}} = \mathbb{B}^P_{\mu\nu}L_{\nu\underline{i}} \quad \forall \mu, \underline{i}$
    end for
    for aux-batch2 $\geq$ aux-batch1 **do**
        read $\tilde{\underline{\mathbb{B}}}^Q_{\mu\underline{i}}(-\mathrm{i}\tau) \quad \forall \underline{i}, \mu, Q \in$ aux-batch2
        for $B \in \mathbf{B} = \{B_x, B_y, B_z\}$ **do**
            for $P \in$ aux-batch1 **do** ▷ parallel
                $\big(\tilde{\mathbb{B}}^P_{\mu\underline{i}}\big)^B(\mathrm{i}\tau) = \overline{\mathbb{B}}^P_{\nu\underline{i}}\overline{G}^B_{0,\nu\mu}(\mathrm{i}\tau) \quad \forall \mu, \underline{i}$
            end for
            for $P \in$ aux-batch1 **do**
                for $Q \in$ aux-batch2 **do**
                    $X^B_{0,PQ}(\mathrm{i}\tau) \mathrel{+}= \big(\tilde{\mathbb{B}}^P_{\mu\underline{i}}\big)^B(\mathrm{i}\tau)\tilde{\underline{\mathbb{B}}}^Q_{\mu\underline{i}}(-\mathrm{i}\tau)$
                end for
            end for
        end for
    end for
end for

2 Batching-Results: Linear Alkanes

Tab. S1 provides the number of batches corresponding to Fig. 7.

Table S1: Number of auxiliary function batches for various intermediates for linear alkanes of increasing size. Calculations were carried out on a compute node with 250 GB of RAM.

Quantity	Number of aux-batches				
	$N = 1690$	$N = 1970$	$N = 2250$	$N = 2530$	$N = 2810$
$\mathbf{X}_0(\mathrm{i}\tau)$	1	1	1	1	3
$\mathbf{X}_0^{\mathbf{B}}(\mathrm{i}\tau)$					
$\frac{\partial \mathbf{X}_0(\mathrm{i}\tau)}{\partial \underline{\mathbf{G}}_0(-\mathrm{i}\tau)}\frac{\partial \underline{\mathbf{G}}_0(-\mathrm{i}\tau)}{\partial \mathbf{B}}$	1	3	4	5	7
$\frac{\partial \mathbf{X}_0(\mathrm{i}\tau)}{\partial \overline{\mathbf{G}}_0(\mathrm{i}\tau)}\frac{\partial \overline{\mathbf{G}}_0(\mathrm{i}\tau)}{\partial \mathbf{B}}$	1	1	1	3	3
$\frac{\partial \mathbf{X}_0(\mathrm{i}\tau)}{\partial \mathbb{B}}\frac{\partial \mathbb{B}}{\partial \mathbf{B}}$	1	1	1	3	5
$\mathbf{\Sigma}(\mathrm{i}\tau)$	1	1	1	1	2
$\mathbf{\Sigma}^{\mathbf{B}}(\mathrm{i}\tau)$					
$\frac{\partial \mathbf{\Sigma}(\mathrm{i}\tau)}{\partial \tilde{\mathbf{W}}_{\mathrm{c}}(-\mathrm{i}\tau)}\frac{\partial \tilde{\mathbf{W}}_{\mathrm{c}}(-\mathrm{i}\tau)}{\partial \mathbf{B}}$	1	1	1	2	2
$\frac{\partial \mathbf{\Sigma}(\mathrm{i}\tau)}{\partial \mathbf{G}_0(\mathrm{i}\tau)}\frac{\partial \mathbf{G}_0(\mathrm{i}\tau)}{\partial \mathbf{B}}$	1	1	1	1	1
$\frac{\partial \mathbf{\Sigma}(\mathrm{i}\tau)}{\partial \mathbb{B}}\frac{\partial \mathbb{B}}{\partial \mathbf{B}}$	1	1	1	2	2

3 Timings: Dense vs. Sparse Implementation

NMR shieldings were computed at the RPA@HF level of theory using the cc-pwCVDZ basis set with the corresponding RI basis set for linear alkanes. The computations were carried out using the ω-CDD-RI-RPA-NMR method, that is, the atomic-orbital RPA NMR formulation with an attenuated Coulomb RI metric and Cholesky decomposed density type matrices. Tab. S2 compares the timings for the computations using dense matrix algebra, denoted as 'Dense', and using sparse matrix algebra, denoted as 'Sparse'. The computations were carried out on a compute node with AMD EPYC 7302 processors using 64 threads, 250 GB of RAM, and 1.7 TB of disk space. For dense matrix algebra routines the Intel Math Kernel Library (version 2022.0.0) was employed.

Table S2: Timings for the computation of the RPA correlation part within the computation of NMR shieldings at the RPA@HF/cc-pwCVDZ level of theory for the ω-CDD-RI-RPA-NMR method using dense and sparse matrix algebra for linear alkanes.

System	Wall Time [s]	
	Dense	Sparse
C_5H_{12}	61.34	72.38
$C_{10}H_{22}$	312.81	309.13
$C_{20}H_{42}$	1775.70	1418.08
$C_{30}H_{62}$	5689.82	3415.05

4 Isotropic NMR shielding constants for $(DNA)_4$/cc-pwCVDZ

Table S3: Isotropic NMR shielding constants for $(DNA)_4$ computed using RPA@HF (RPA based on a preceding HF calculation) and the cc-pwCVDZ basis set with the corresponding RI basis set.

Nucleus	Shieldings [ppm]
H	1.57833
H	1.390977

Table S3: (continued)

H	0.74982
O	39.625412
C	1.525101
C	18.766431
H	4.234651
C	8.601825
H	1.334232
N	5.214792
O	17.11874
H	0.619695
C	17.137739
C	13.991661
O	0.76482
H	0.011777
H	-0.037214
N	3.382744
H	0.71742
C	-0.030678
O	-4.852926
H	0.164014
C	-1.494732
H	0.8372
H	0.626706

Table S3: (continued)

C	-0.995834
C	0.181645
H	0.264816
H	1.446535
H	1.880048
H	1.201433
O	22.899628
H	0.90182
C	-0.18823
C	1.699186
C	17.939165
H	4.876313
O	5.865786
O	-0.517268
O	3.17016
C	8.729024
H	1.672155
N	9.706986
O	18.694527
P	-5.558484
C	17.851583
C	15.038718
O	-1.16659

Table S3: (continued)

H	0.551018
H	0.292424
N	3.527311
H	1.234812
C	-0.122849
O	-4.047136
H	0.476733
C	-1.016173
H	1.023262
H	0.81029
C	-1.005319
C	0.469242
H	0.401737
H	0.972414
H	1.431564
H	1.057215
O	28.595151
H	0.713146
C	0.058148
C	1.184723
C	17.507499
H	5.095655
O	5.81443

Table S3: (continued)

O	-1.839941
O	5.196133
C	8.006601
H	1.577159
N	11.366697
O	19.340224
P	-6.552366
C	18.108446
C	15.040221
O	-0.071245
H	0.736576
H	0.389637
N	3.613857
H	1.191251
C	0.015605
O	-3.912513
H	0.634324
C	-1.013577
H	0.765568
H	0.746337
C	-0.87146
C	0.277107
H	0.422746

Table S3: (continued)

H	0.708284
C	0.121936
O	6.030582
O	-1.702112
O	7.620857
P	-6.168668
H	0.550693
O	-0.527313
H	0.730181
H	0.358968
O	0.543564
H	0.208021
C	0.101372
H	0.601108
C	-0.243286
H	0.405819
H	0.393301
C	-0.907799
C	0.163644
O	-3.917446
H	0.707194
O	15.168253
C	-1.382187

Table S3: (continued)

C	13.889823
H	2.942117
N	3.281975
H	1.113149
N	12.03376
O	29.206439
C	17.825503
C	15.360425
C	6.329633
H	0.608848
H	0.514207
C	0.423383
H	0.392887
H	2.627137
H	-1.669095
N	23.600778
N	14.486347
H	1.437691
C	-12.18866
C	19.393901
C	10.615719
N	10.929766
N	35.457777

Table S3: (continued)

H	0.541916
C	16.505611
C	14.777031
H	0.531465
N	4.828071
H	0.67236
O	0.971618
H	-0.012738
H	-0.085093
H	5.723445
H	3.634883
C	-1.569247
H	0.549683
H	0.996448
C	-0.036606
O	-4.625606
H	0.132045
N	3.035753
N	8.401628
H	3.482241
C	0.35842
C	-1.211749
H	0.295052

Table S3: (continued)

C	17.904118
C	22.012332
H	0.649918
C	0.083592
C	7.155296
N	14.804725
N	38.438202
H	2.121572
O	5.909475
O	-1.372144
O	8.308139
C	18.475192
C	17.977088
P	-5.856548
N	6.676367
H	1.644376
O	-0.548599
H	0.368341
H	0.672384
H	6.422013
H	6.702008
C	-0.441486
H	1.286438

Table S3: (continued)

H	1.551719
C	0.094695
O	-3.279313
H	0.656462
N	20.344932
N	17.348439
H	4.026186
C	0.978224
C	-0.8653
H	0.619598
C	22.681794
C	22.697891
H	0.765969
C	0.563298
C	10.044622
N	13.580254
N	41.966413
H	2.404027
O	5.971144
O	-1.176067
O	8.216446
C	20.655167
C	18.88493

Table S3: (continued)

P	-5.806898
N	7.221697
H	1.41152
O	-0.545968
H	0.470987
H	0.947656
C	-0.63689
H	0.879467
H	0.463376
C	0.180698
O	-3.046955
H	0.772728
C	0.383694
C	-0.789458
H	0.269318
C	-0.012608
H	0.427743
O	-1.47565
O	5.690395
O	2.276213
P	-6.347167
H	0.058347
O	-0.367606

Table S3: (continued)

H	0.227289
H	0.611955
O	0.100699
H	-0.239016
C	-0.088877
H	0.312979
C	-0.739927
H	-0.609891
H	-0.434058
C	-1.201207
C	-0.582964
O	-3.537079
H	-0.133302
C	-2.177435
H	-0.451347
N	4.87628
C	14.281054
N	8.532255
H	2.11225
C	14.638668
N	34.43981
C	21.046854
C	4.679381

Table S3: (continued)

N	44.749026
C	7.53773
H	-10.798186
N	43.585368
H	9.508841
